# A Photochemical Reaction in Different Theoretical Representations

**DOI:** 10.1021/acs.jpca.1c09604

**Published:** 2022-02-14

**Authors:** Lea M. Ibele, Basile F. E. Curchod, Federica Agostini

**Affiliations:** † Department of Chemistry, Durham University, Durham DH1 3LE, United Kingdom; ‡ Université Paris-Saclay, CNRS, Institut de Chimie Physique UMR8000, 91405 Orsay, France

## Abstract

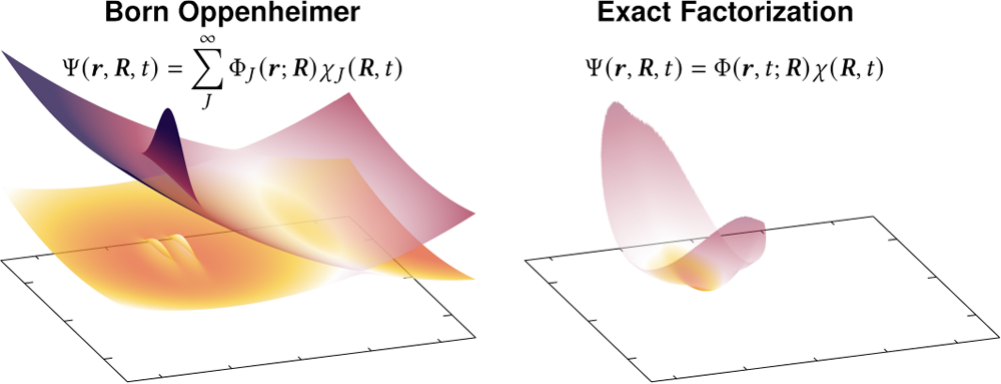

The Born–Oppenheimer picture has forged our representation and interpretation of photochemical processes, from photoexcitation down to the passage through a conical intersection, a funnel connecting different electronic states. In this work, we analyze a full in silico photochemical experiment, from the explicit electronic excitation by a laser pulse to the formation of photoproducts following a nonradiative decay through a conical intersection, by contrasting the picture offered by Born–Oppenheimer and that proposed by the exact factorization. The exact factorization offers an alternative understanding of photochemistry that does not rely on concepts such as electronic states, nonadiabatic couplings, and conical intersections. On the basis of nonadiabatic quantum dynamics performed for a two-state 2D model system, this work allows us to compare Born–Oppenheimer and exact factorization for (i) an explicit photoexcitation with and without the Condon approximation, (ii) the passage of a nuclear wavepacket through a conical intersection, (iii) the formation of excited stationary states in the Franck–Condon region, and (iv) the use of classical and quantum trajectories in the exact factorization picture to capture nonadiabatic processes triggered by a laser pulse.

## 1 Introduction

Our way of picturing molecules and chemical processes has been greatly shaped by the Born–Oppenheimer approximation, the assumption that the motion of electrons and nuclei can be treated separately in a molecule.^1,2^ The discussion of chemical structures, properties, and reactivity usually intrinsically assumes that the molecule remains in a given electronic eigenstate or, in other words, that electrons can adapt instantaneously to any nuclear motion, which is a direct consequence of the Born–Oppenheimer approximation. Photochemistry inherently goes beyond this picture because photon absorption by a molecule makes transitions to different electronic states possible.^3−9^ The subsequent out-of-equilibrium evolution of the excited molecule usually means that regions where one or more electronic states come close in energy can be visited. When the nuclear dynamics drives the molecule into such regions, the Born–Oppenheimer approximation breaks down. The *nonadiabatic* coupling between nuclear and electronic motions cannot be neglected anymore, and one needs to accept that nuclear motion can lead to a change of electronic states.^10^

A legitimate strategy to move beyond the Born–Oppenheimer approximation would be to try to supplement the Born–Oppenheimer picture with more electronic eigenstates and account for their mutual couplings mediated by nuclear motion. Importantly, this post-Born–Oppenheimer picture, often called the Born–Huang representation,^11^ relies on the use of quantities originally defined within the framework of the Born–Oppenheimer approximation, such as the potential energy surfaces obtained from the electronic Schrödinger equation for fixed nuclear degrees of freedom, in a context that is at the opposite of their initial raison d’être: strong electron–nuclear couplings.^12^ This post-Born–Oppenheimer picture is at the heart of our way to regard photochemical processes, and the vocabulary used for such processes is intrinsically shaped by Born–Oppenheimer concepts: potential energy surfaces (eigenvalues from the time-independent Schrödinger equation),^4^ conical intersections (points of degeneracy between two adiabatic potential energy surfaces),^13,14^ the Berry phase (a phase picked up in the adiabatic representation when encircling a conical intersection),^15,16^ and the transition dipole moment (the electric dipole moment for the transition between two electronic states). Within this picture, the molecular wave function is expanded in the adiabatic basis, that is, using the set of time-independent eigenstates of the (electronic) Born–Oppenheimer Hamiltonian. An alternative choice for this basis expansion is to use the diabatic representation, which diagonalizes, when possible, the nuclear kinetic energy operator. In the diabatic representation, the electronic states preserve their character upon varying the nuclear configuration. Conical intersections and the Berry phase are not present in this representation because the electron–nuclear coupling is mediated here via off-diagonal elements of the electronic Hamiltonian matrix. For systems with more than two electronic states, where diabatic states might not be defined, one needs to rely on quasi-diabatic states that correspond to a set of electronic states that minimize the nonadiabatic couplings.

An exact theoretical description of a photochemical process necessitates the solution of the full time-dependent Schrödinger equation, a task that is possible only for the smallest molecular systems. In the vast majority of cases, the in silico simulation of excited-state (or nonadiabatic) molecular dynamics is performed under the (post-) Born–Oppenheimer picture. In the simplest case, the molecule of interest is considered to be described by its ground vibrational and electronic states, an external time-dependent field (for example, a laser pulse) is applied to the molecule, and the light–matter interaction induces an electronic excitation leading to the formation of a nuclear wavepacket in an excited electronic state. This nuclear wavepacket subsequently evolves and can branch in regions of strong nonadiabaticity, leading to its (possibly partial) transfer to different electronic states, a nonradiative decay, until the ground state is potentially reached. The out-of-equilibrium dynamics experienced by the molecule during this overall process can make it explore regions of the nuclear configuration space that would not be thermally accessible from the ground electronic state, leading to the formation of so-called photoproducts.^17−21^ The formation of these photoproducts can be triggered in the excited electronic states or during subsequent athermal dynamics in the ground electronic state following the nonradiative decay.^22,23^ Accessing the photochemistry of molecules in their full dimensionality usually requires approximations for the dynamics, which are in many cases based on representing the nuclear wave functions through trajectory basis functions or substituting it with swarms of classical trajectories. (See refs (24−26) for examples of such strategies.) A consequence of the Born–Oppenheimer picture is that one needs to find strategies to account for nonadiabatic effects in these approximate methods and, more specifically, to describe the branching of nuclear wavepackets between different electronic states. A plethora of ideas and algorithms were proposed, where coupled or uncoupled trajectories would hop, evolve on mean-field surfaces, spawn, or clone other trajectories.^27^

In this work, we propose to explore the different steps of a typical photochemical experiment described above with an alternative representation of the coupled electron–nuclear dynamics of a molecule: the exact factorization.^28,29^ The exact factorization introduces a framework exempt from the ideas emanating from the Born–Oppenheimer picture; there is no mention of electronic states or static potential energy surfaces. Instead, the exact factorization depicts the dynamics of a molecular system by a nuclear wave function whose dynamics is dictated by a single time-dependent vector^30^ and scalar^31^ potential. Earlier work^30,32−34^ showed that this formalism changes our way of representing nonadiabatic molecular dynamics. In particular, the concepts of the transition between electronic states, conical intersections, and the (topological) Berry phase do not appear in the exact factorization. Hence, there is a real curiosity in unraveling how the exact factorization would describe a full photochemical experiment for a 2D two-state molecular model, from photoexcitation with a laser pulse to the formation of photoproducts, and in comparing this picture to the more conventional Born–Oppenheimer representation. In addition, these simulations will allow us to shed light on other interesting aspects of an in silico photochemical experiment, such as (i) the effect of the Condon approximation, (ii) the analysis of the dynamics using representation-free quantities, and (iii) the use of classical and quantum trajectories to depict the entire nuclear dynamics during a photochemical process.

This article is organized as follows. We propose in section 2 a brief review of the Born–Oppenheimer (section 2.1) and exact-factorization (section 2.2) pictures to describe coupled electron–nuclear dynamics such as that observed in a photochemical process. We then define our two-state 2D model Hamiltonian and the light–matter interaction Hamiltonian with and without the Condon approximation in section 2.3. In the same section, we also highlight some important considerations on our model and provide the computational details. We then present the results of our in silico photochemical experiment in section 3, starting with the more conventional Born–Oppenheimer picture and then moving to the exact factorization. We also discuss the use of classical and quantum trajectories within the exact factorization formalism. Our conclusions are finally stated in section 4.

## 2 Methods

The evolution of the time-dependent wave function describing the state of a molecule, Ψ(**r**, **R**, *t*), follows the time-dependent Schrödinger equation
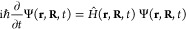
1where the full Hamiltonian operator *Ĥ*(**r**, **R**, *t*) includes the nuclear kinetic energy *T̂*_n_(**R**), a Born–Oppenheimer Hamiltonian *Ĥ*_BO_(**r**, **R**), and an external (time-dependent) potential *V̂*(**r**, **R**, *t*):
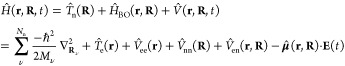
2**R** and **r** are collective variables for the coordinates of the nuclei and electrons forming the molecule. The summation for the nuclear kinetic energy runs over all *N*_n_ nuclei, each labeled by ν and with corresponding mass *M*_ν_. The Born–Oppenheimer Hamiltonian consists of the electronic kinetic energy *T̂*_e_(**r**), the electron–electron *V̂*_ee_(**r**), the nucleus–nucleus *V̂*_nn_(**R**), and the electron–nucleus *V̂*_en_(**r**, **R**) interaction. In the dipole approximation, the effect of an external time-dependent electric field **E**(*t*) coupled to the electric dipole moment operator **μ̂**(**r**, **R**) is encoded in *V̂*(**r**, **R**, *t*).

### 2.1 Born–Oppenheimer Picture of Nonadiabatic Dynamics

Within the Born–Oppenheimer approximation, the molecular wave function Ψ(**r**, **R**, *t*) is approximated as a single product of a time-independent electronic wave function, Φ_*J*_(**r**; **R**), that is the *J*th solution of the time-independent electronic Schrödinger equation and a corresponding time-dependent nuclear wave function, χ_*J*_(**R**, *t*):
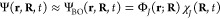
3This approximation restricts the dynamics of the nuclear wave function χ_*J*_(**R**, *t*) to the adiabatic electronic state *J*. In other words, the nuclear wave function evolves adiabatically in a given electronic state, and nothing can trigger a change in the electronic state with the Born–Oppenheimer approximation. To move away from an adiabatic representation and account for nonadiabatic effects, one needs to move to a different representation of the molecular wave function called the Born–Huang expansion. In the Born–Huang representation, the molecular wave function is written as
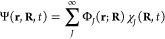
4Hence, the Born–Oppenheimer approximation has been corrected via an infinite sum over all electronic states *J* leading to an in principle exact expansion of the molecular wave function. By inserting this expression into the molecular Schrödinger equation (eq 1), we can (after left multiplication by Φ_*I*_(**r**; **R**) and integration over all electronic coordinates **r**) obtain a set of equations of motion for the nuclear amplitudes (χ_*I*_(**R**, *t*)). (We assume a general form for the nuclear amplitudes that could include any phases picked up during the dynamics as a function of **R**.)
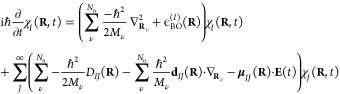
5The first two terms on the right-hand side denote the nuclear kinetic and potential energy associated with the adiabatic dynamics of the nuclear wave function in electronic state *I*. We can picture the nuclear amplitude associated with state *I* to be evolving on the (static) potential energy surface ϵ_BO_^(*I*)^(**R**) = ⟨Φ_*I*_(**R**)|*Ĥ*_BO_(**R**)|Φ_*I*_(**R**)⟩_**r**_. We note that ⟨···⟩_**r**_ indicates integration over all electronic coordinates **r**. The three last terms on the right-hand side of eq 5, neglected within the Born–Oppenheimer adiabatic approximation, are responsible for the possible transfer of nuclear amplitude between electronic states: *D*_*IJ*_(**R**) = ⟨Φ_*I*_(**R**)|∇_**R**_^2^|Φ_*J*_(**R**)⟩_**r**_ represents the second-order nonadiabatic couplings, **d**_*IJ*_(**R**) = ⟨Φ_*I*_(**R**)|∇_**R**_|Φ_*J*_(**R**)⟩_**r**_ represents the first-order nonadiabatic coupling vectors, and the final term determines the light–matter interaction with the external time-dependent electric field within the dipole approximation. The (de)excitation process by an electric field **E**(*t*) is mediated by the transition dipole moment **μ**_*IJ*_(**R**) = ⟨Φ_*I*_(**R**)|**μ̂**(**R**)|Φ_*J*_(**R**)⟩_**r**_ of the molecule. We note that **d**_*II*_(**R**) = 0 for real electronic wave functions, and *D*_*II*_(**R**) and **μ**_*II*_(**R**) are not necessarily zero.

### 2.2 Exact Factorization Picture of Nonadiabatic Dynamics

Instead of expressing the molecular wave function as an infinite sum over time-independent electronic eigenfunctions and time-dependent nuclear amplitudes, the exact factorization proposes a subtle alternative:
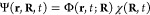
6This exact representation of the molecular wave function relies on a single product composed of a nuclear wave function χ(**R**, *t*) and an electronic wave function Φ(**r**, *t*; **R**), both time-dependent. Importantly, the electronic wave function does still depend parametrically on **R**. Using the partial normalization condition, ∫ d**r**|Φ(**r**, *t*; **R**)|^2^ = 1 *∀***R**, *t*, ensures that |χ(**R**, *t*)|^2^ will reproduce the nuclear density as obtained from Ψ(**r**, **R**, *t*) at all times. Both χ(**R**, *t*) and Φ(**r**, *t*; **R**) are uniquely defined up to a phase factor of exp[(i/*ℏ*)θ(**R**, *t*)] for any choice of the gauge function θ(**R**, *t*). In general, θ(**R**, *t*) does not have any physical meaning in the sense that observable properties do not depend on θ(**R**, *t*). Note that Ψ(**r**, **R**, *t*) = χ(**R**, *t*) Φ(**r**, *t*; **R**) = χ̃(**R**, *t*) Φ̃(**r**, *t*; **R**) if one defines χ̃(**R**, *t*) = exp[−(i/*ℏ*)θ(**R**, *t*)]χ(**R**, *t*) and Φ̃(**r**, *t*; **R**) = exp[(i/*ℏ*)θ(**R**, *t*)]Φ(**r**, *t*; **R**). The partial normalization condition also holds for the gauge-transformed electronic wave function. Once this gauge freedom is fixed by imposing a choice of phase, eq 6 is unique.

Because now both electronic and nuclear wave functions depend on time, we get (after insertion into the time-dependent Schrödinger equation) a set of coupled equations of motion:
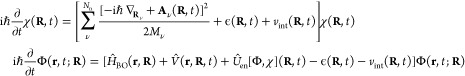
7These equations of motion introduce two new potentials within the framework of the exact factorization: the time-dependent vector potential, **A**_ν_(**R**, *t*) = ⟨Φ(*t*; **R**)|−iℏ∇_**R**_ν__Φ(*t*; **R**)⟩_**r**_, and the time-dependent potential energy surface consisting of the two scalar potentials, ϵ(**R**, *t*) = ⟨Φ(*t*; **R**)|*Ĥ*_BO_(**R**) + *Û*_en_[Φ, χ](**R**, *t*) – iℏ∂_*t*_|Φ(*t*;**R**)⟩_**r**_ and *v*_int_(**R**, *t*) = ⟨Φ(*t*; **R**)|*V̂*(**R**, *t*)|Φ(*t*; **R**)⟩_**r**_. The electron–nuclear coupling operator, *Û*_en_[Φ, χ](**R**, *t*), is explicitly dependent on the nuclear wave function χ(**R**, *t*) and through the time-dependent vector potential, **A**_ν_(**R**, *t*), also implicitly contains the electronic wave function Φ(**r**, *t*; **R**):
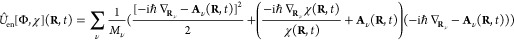
8It is worth mentioning here that the nuclear momentum field computed from the molecular wave function Ψ(**r**, **R**, *t*) can be decomposed as the sum of a curl-free contribution, which is related to the phase of the nuclear wave function χ(**R**, *t*) and (in general) a non-irrotational contribution, which is given by the time-dependent vector potential **A**_ν_(**R**, *t*). The relationship between the nuclear momentum field and the vector potential will be used in Appendix B, where we illustrate the procedure employed to introduce a trajectory-based description of the nuclear dynamics within the framework of the exact factorization. In particular, we clarify the difference between quantum^35−37^ and classical trajectories^38,39^ used in the numerical studies proposed in section 3.2.

### 2.3 Presentation of the Two-State Two-Dimensional Model

#### 2.3.1 Computational Details

We used for this study a 2D two-state molecular model. In the diabatic representation, the general form of the Hamiltonian is given by
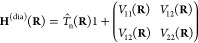
9with the following diabatic electronic energies
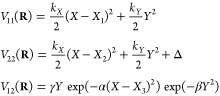
10using **R** = (*X*, *Y*). The parameters *k*_*X*_ = 0.02 *E*_h_*a*_0_^–2^, *k*_*Y*_ = 0.1 *E*_h_*a*_0_^–2^, Δ = 0.01*E*_h_, γ = 0.01 *E*_h_*a*_0_^–1^, α = 3*a*_0_^–2^, β = 1.5*a*_0_^–2^, *M*_*X*_ = 20 000.0*m*_e_, and *M*_*Y*_ = 6667.0*m*_e_ are based on refs (34) and (40), and we set here *X*_1_ = 6*a*_0_, *X*_2_ = 2*a*_0_, and *X*_3_ = 3.875*a*_0_. The two states are taken to be within the singlet manifold, so henceforth the adiabatic states will be labeled S_0_ (ground electronic state) and S_1_ (first excited electronic state).

We propagated nuclear wavepackets with numerically exact quantum dynamics, using for the initial state a Gaussian function with widths σ_*X*_ = 0.15*a*_0_ and σ_*Y*_ = 0.197*a*_0_ initialized in the adiabatic ground electronic state with zero initial nuclear momentum. The nuclear wave function is initially positioned at the Franck–Condon point **R**_init_ = (2.0, 0.0)*a*_0_. We note that because of the weak anharmonicity, the Franck–Condon point in the adiabatic representation coincides with the minimum of *V*_22_(**R**).

The external time-dependent electric field of a laser pulse under study here is given by
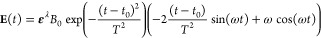
11with amplitude *B*_0_ = 0.065*ℏ*(*ea*_0_)^−1^, centered at *t*_0_ = 350.0*ℏE*_h_^–1^, with duration *T* = 141.421356 *ℏE*_h_^–1^ and frequency ω = 0.15*E*_h_*ℏ*^–1^. **ε**^λ^ is the polarization vector, set to  for all calculations. (The parameters of the external time-dependent electric feld were chosen to resemble a typical femtosecond pulse in terms of amplitude and duration, and the frequency was adjusted to be in resonance with the S_0_ → S_1_ transition at the Franck–Condon point.) According to Maxwell’s equations, the external electric field is the time derivative of a (purely time-dependent, in the long-wavelength approximation) vector potential. Equation 11 is derived from such a vector potential assuming a Gaussian-shaped laser pulse, as discussed in ref (41). The transition dipole moment in the diabatic representation is chosen to be **μ**_12_(**R**) = (*f*_*X*_(*X* – *X*_0_), *f*_*Y*_(*Y* – *Y*_0_)), with *f*_*X*_ = 0.2*e*, *f*_*Y*_ = 2.0*e*, *X*_0_ = −1.0*a*_0_, and *Y*_0_ = −0.5*a*_0_. In the Condon approximation, **μ**_12_(**R** = **R**_init_) is used. The diagonal elements of the dipole operator are set to zero.

The full time-dependent Schrödinger equation is solved numerically in the diabatic representation employing a split-operator formalism^42,43^ with a time step of 0.01*ℏE*_h_^–1^. A spatial grid of 800 points per coordinate is used over the ranges of *X* ∈ [0.0, 8.0]*a*_0_ and *Y* ∈ [−2.0, 2.0]*a*_0_. Diabatic quantities are consequently transformed to give all of the respective exact-factorization quantities of interest, namely, the time-dependent vector potential and the time-dependent potential energy surface. The expressions used for this transformation are given in Appendix A. To avoid numerical issues in the conversion, the time-dependent vector potential and the time-dependent potential energy surface are calculated only for regions of the nuclear configuration space where the nuclear density is larger than 10^–8^.

Phase factor θ(**R**, *t*) is absorbed here into phase *S*(**R**, *t*) of the nuclear wave function, χ(**R**, *t*) = |χ(**R**, *t*)| exp[(i/*ℏ*)*S*(**R**, *t*)], and the gauge is chosen so that the nuclear wave function is real and non-negative, i.e., χ(**R**, *t*) = |χ(**R**, *t*)|∀**R**, *t*. The exact nuclear density is determined as the sum of the squared moduli of the diabatic nuclear wave functions, and its positive square root yields the nuclear wave function χ(**R**, *t*) in the chosen gauge.

#### 2.3.2 Some Considerations for the Proposed Model

**Figure 1:**
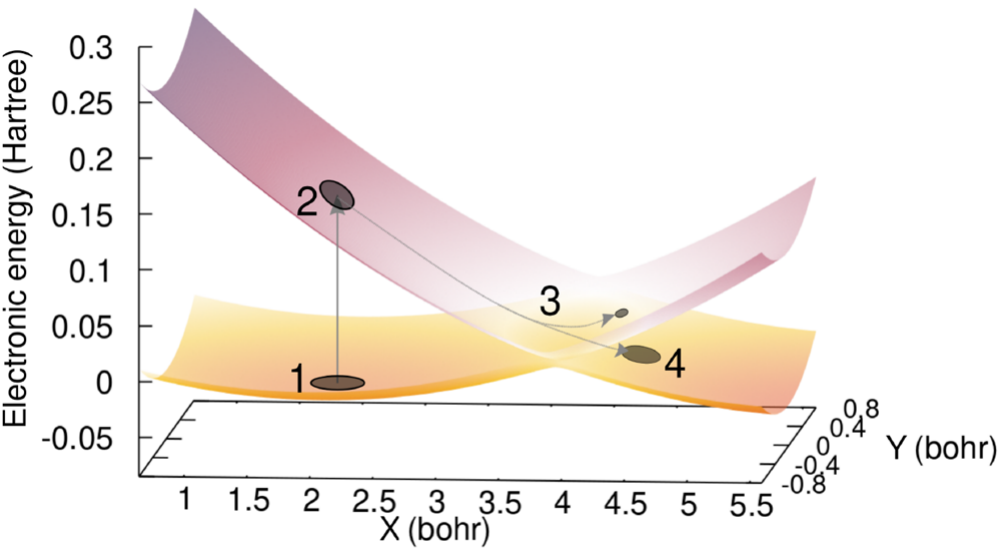
Schematic representation of the in silico photochemical experiment discussed in this work. The adiabatic potential energy surfaces for the ground (S_0_) and excited (S_1_) electronic states of the model are represented with a color map that shows positive electronic energies in purple and negative values in yellow/orange tones. The black circles and arrows indicate the different steps of the photochemical experiment: (1) initial state of the system in the Franck–Condon region, (2) photoexcitation from S_0_ to S_1_ triggered by a laser pulse, (3) relaxation through a conical intersection, and (4) formation of photoproducts.

In this work, we propose to simulate explicitly all of the steps in a photochemical process for the model system discussed above, as schematically represented in Figure 1. The model system consists of two 2D parabolas shifted in the *X* direction and in energy. At time *t* = 0, our molecular system is in its ground vibrational state, in the Franck–Condon region of the electronic ground state (point (1) in Figure 1). Then, a part of the initial wave function is excited to the S_1_ excited electronic state via the coupling of the molecule with an explicit ultrashort laser pulse whose frequency is in resonance with the S_0_-to-S_1_ transition (point (2) in Figure 1). This photoexcitation generates a nuclear wavepacket in the excited electronic state, which will relax toward the conical intersection—a point of configuration space where the ground and first electronic states are degenerate—and funnel through it (point (3) in Figure 1). At this point, the nuclear wavepacket experiences branching, preserving some of its amplitude in the excited electronic state while a good part of it has transferred back to the ground state as a result of nonadiabatic effects, evolving now as a photoproduct on the ground electronic state (point (4) in Figure 1).

We stress at this stage that the schematic representation of the photochemical experiment depicted in Figure 1 is deeply rooted in a (post-) Born–Oppenheimer picture. In section 3.1, we will discuss the details of the dynamics in terms of time-dependent nuclear wavepackets and static potential energy surfaces, thus adopting a Born–Oppenheimer vocabulary. In section 3.2, we will revisit this analysis from the perspective of the exact factorization, where the overall dynamics is examined in terms of a time-dependent nuclear wavepacket evolving according to a single time-dependent vector and scalar potential.

**Figure 2:**
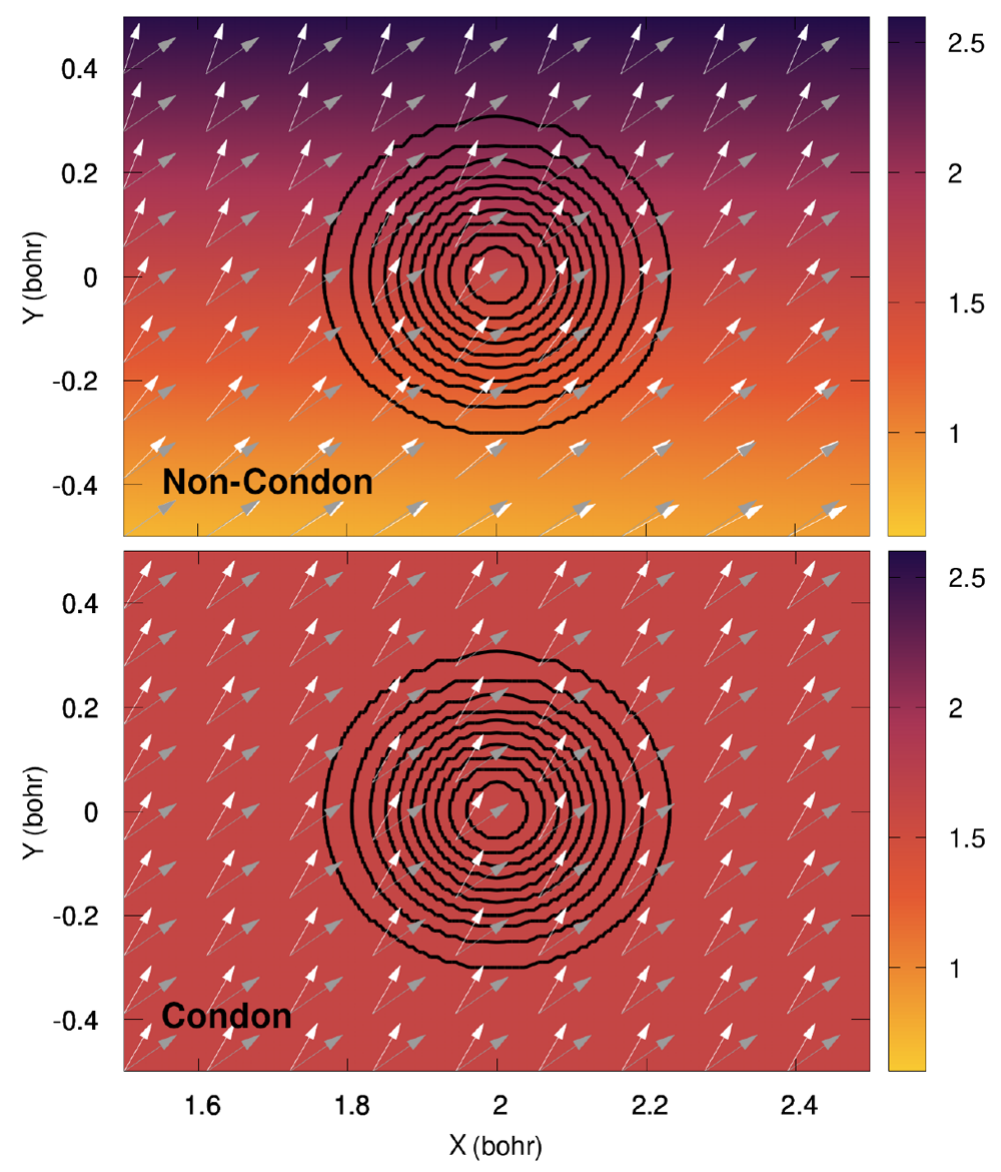
Representation of the transition dipole moment (white arrows give its direction and color map its intensity in *ea*_0_) in the non-Condon case (upper panel) and the Condon case (lower panel) around the Franck–Condon region. The gray arrows indicate the polarization vector of the time-dependent electric field. The black contour lines show the nuclear density of the initial wave function at time *t* = 0.

Another aspect that requires a comment at this stage is the coupling of an external time-dependent electric field, here a laser pulse, to a molecule. As described above, we use a semiclassical approach to couple an external time-dependent electric field to the molecular dipole operator. When introducing a given basis for our electronic states, once more preserving here a Born–Oppenheimer/Huang picture, we obtain couplings between the time-dependent electric field and the transition dipole moment between the pair of electronic states considered (here S_0_ and S_1_), **μ**_12_(**R**). The magnitude and direction of the transition dipole moment depend on the nuclear position, as depicted by the color map and white arrows in the upper panel of Figure 2. This **R** dependence of the transition dipole moment implies that, within the long-wavelength approximation, the time-dependent electric field *cannot* be considered to be always aligned with the transition dipole moment (gray arrows in Figure 2, symbolizing the electric-field polarization vector), and one has to take the scalar product between the two quantities. The situation in which we account for the explicit **R** dependence of the transition dipole moment will now be referred to as non-Condon. The Condon approximation proposes to consider the transition dipole moment as a constant, set to its value at the Franck–Condon point, **μ**_12_(**R**_FC_) (lower panel of Figure 2). Hence, only within the Condon approximation could we consider that the time-dependent electric field is always polarized along the transition dipole moment for all nuclear configurations (which is not the case in the present work).

In the non-Condon case, the strength of the coupling between the molecular system and the time-dependent electric field, given by
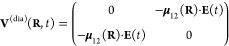
12depends on **R** as well. Conversely, in the Condon approximation the relative orientation of the transition dipole moment and the polarization vector of the time-dependent electric field is constant, leading to an **R**-independent coupling term in the Hamiltonian.

An additional topic that our work addresses is the representation of an in silico photochemical experiment using only the concept of nuclear trajectories, which will allow us to open a broader discussion of the steps toward excited-state molecular dynamics simulations. In section 3.2, we study the use of nuclear trajectories for nonadiabatic dynamics in the context of the exact factorization since well-defined nuclear forces can be identified and provide a clear distinction between classical and quantum trajectories. For the quantum and classical trajectory dynamics, the 1000 initial nuclear positions are sampled from the probability density given by the initial nuclear wave function. Special care needs to be taken when selecting the initial nuclear momenta. While for classical trajectories nuclear positions and momenta can be regarded as independent variables, this is no longer the case for quantum trajectories because their nuclear position and momentum at each time step are related. As shown in Appendix B, the nuclear momentum at time *t* and position **R** is given by the time-dependent vector potential **A**(**R**, *t*). Thus, the 1000 initial nuclear momenta for the sampled initial nuclear positions **R**_0_ are given by **A**(**R**_0_, 0). For classical trajectories, the initial nuclear momenta are sampled from the momentum probability distribution computed from the Wigner transform of the initial nuclear wave function (in position representation). For the propagation of the trajectories, a time step of 10*ℏE*_h_^–1^ is used, and one uses the exact time-dependent vector and scalar potentials as obtained from the quantum dynamics. The time-dependent potentials are not (numerically) available when the nuclear density is small (<10^–8^). Furthermore, classical trajectories are propagated according to the force computed as the negative of the gradient of the time-dependent scalar potential. Therefore, numerical errors due to the calculation of the nuclear gradients can cause the trajectories to move in a region of space where the time-dependent potential is not available. Those trajectories need to be removed from the ensemble. We found that an energy-conservation criterion can be used to remove such unstable trajectories. To this end, we imposed that the classical energy should be conserved within a deviation of 0.01*E*_h_ from the initial value at the end of the laser pulse. A maximum of 51 classical trajectories have been excluded on the basis of this criterion in any of our simulations.

## 3 Results and Discussion

### 3.1 Photochemical Experiment in the Born–Oppenheimer Picture

#### 3.1.1 Nuclear Dynamics

**Figure 3:**
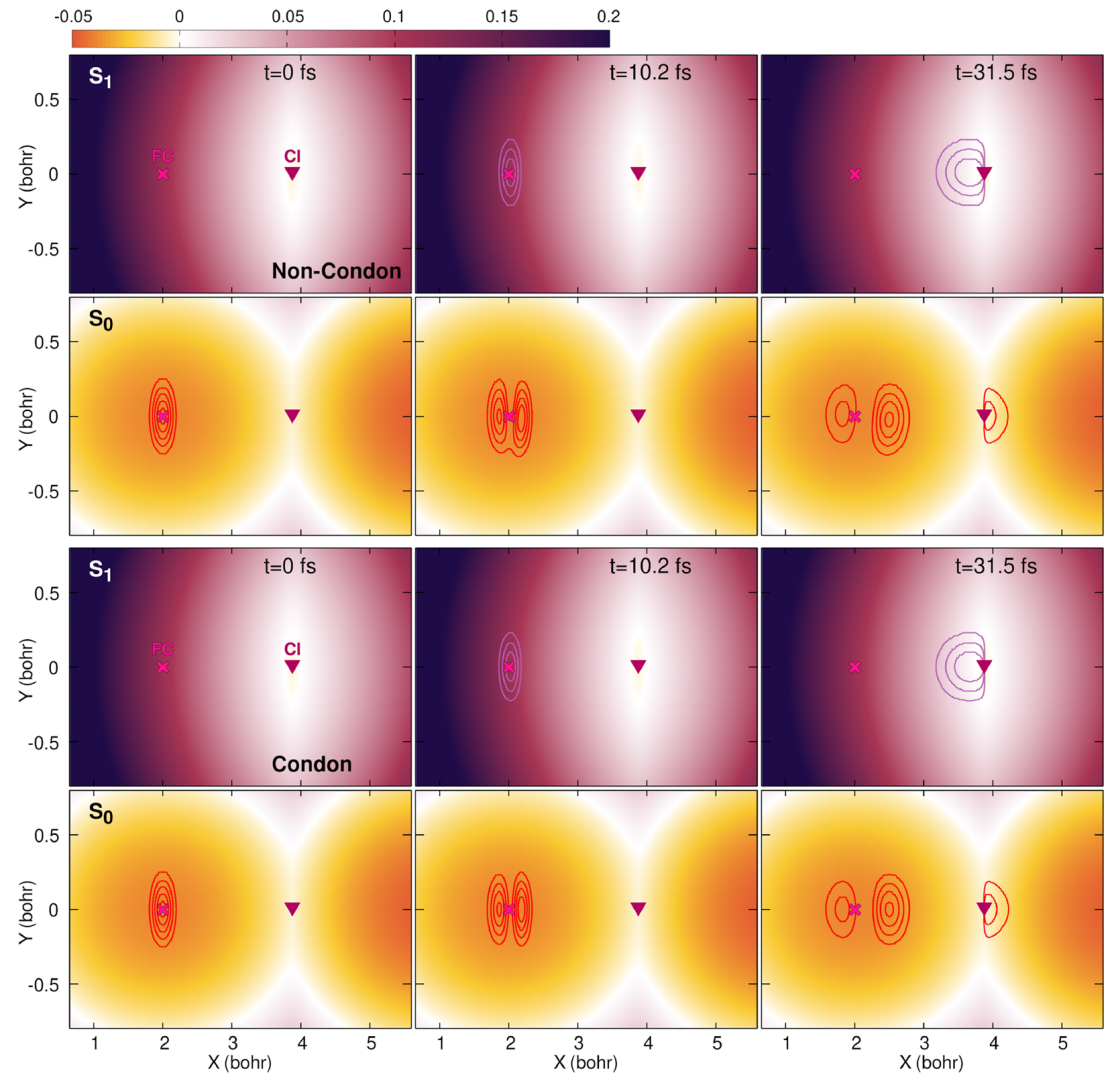
Snapshots of the nonadiabatic quantum dynamics at times 0, 10.2, and 31.5 fs (from left to right). The color maps indicate the (adiabatic) electronic energies of the S_1_ and S_0_ electronic states (see labels) in hartree (*E*_h_). The adiabatic contribution to the nuclear density in each electronic state for the three snapshots is indicated by red  and purple  contour lines. The Franck–Condon point (FC) is indicated by a magenta cross, and the location of the conical intersection (CI) is indicated by a burgundy triangle.

In a Born–Oppenheimer picture, the overall dynamics of an electronically excited molecule is analyzed by following the evolution of nuclear wavepackets on the potential energy surfaces (as depicted schematically in Figure 1). At time *t* = 0, the nuclear wave function is a stationary state in the adiabatic ground state S_0_ (first column in Figure 3). The interaction with the time-dependent external field, consisting of a laser pulse, induces the electronic excitation of part of the original wave function to the first excited state S_1_ (second column in Figure 3). The state created in S_1_ is a nuclear wavepacket, which evolves and decays back to the ground state through the conical intersection (third column in Figure 3).

**Figure 4:**
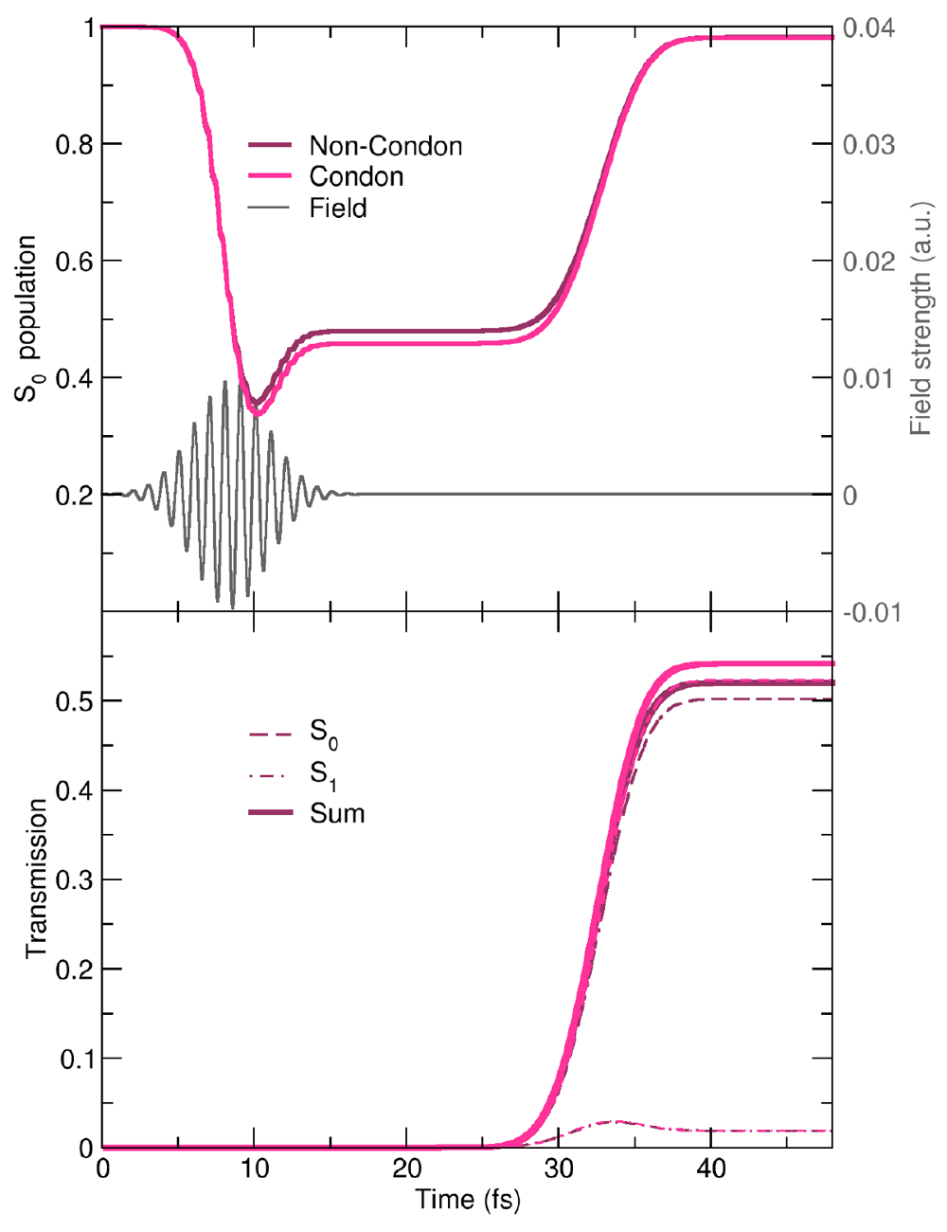
(Top) Time trace of the ground-state (S_0_) population for the non-Condon (purple) and Condon (magenta) quantum dynamics simulations and the strength of the electric component of the laser pulse (gray line) in *E*_h_/*ea*_0_. (Bottom) Time trace of the transmission probability through an ideal barrier at *X* > 3.86*a*_0_ for the non-Condon (purple lines) and Condon simulations (magenta lines). Solid lines indicate the total (S_0_ + S_1_) probability, and the dashed (dotted) lines give the S_0_ (S_1_) population contribution.

First, let us investigate the quantum dynamics obtained in the non-Condon case (upper two rows of panels in Figure 3). The color map shows the potential energy of the S_1_ (top row) and S_0_ (second row from top) states, and the locations of two critical points of the potential energy surfaces—the Franck–Condon point (FC) and the conical intersection (CI)—are marked throughout the plots with a cross and a triangle for reference. Because we are now focusing on the Born–Oppenheimer picture of a photochemical process, the potential energy surfaces of the two adiabatic states do not move or change during the dynamics but can rather be seen as the electronic landscape on the support of which the nuclear wavepackets evolve. The nuclear densities associated with the S_0_ and S_1_ states are superimposed onto the respective potentials in Figure 3 and are indicated as red and purple contour lines, respectively. At time *t* = 0, the complete nuclear density is found in the S_0_ state without any contribution from S_1_. During the photoexcitation by the laser pulse (*t* = 10.2 fs in Figure 3; the time evolution of the laser field is shown in Figure 4), nuclear amplitude is transferred to the excited electronic state. In addition, it can be observed that the nuclear contribution remaining in the ground electronic state is also affected by the action of the laser pulse. Once the short laser pulse is over, the excited portion of the nuclear wavepacket relaxes on the S_1_ potential energy surface, and at *t* = 31.5 fs, it reaches the conical intersection (last column in Figure 3). At this precise moment, part of the nuclear density transfers to the S_0_ electronic state due to the influence of nonadiabatic effects. Interestingly, the portion of the nuclear wavepacket that remained in the Franck–Condon region in S_0_ formed a nodal line as a result of the photoexcitation process. This nodal line is somehow tilted, that is, not parallel to the *Y* axis (red contour lines in the second row, last column panel of Figure 3).

Within the Condon approximation (bottom two rows in Figure 3), the potential energy surfaces remain identical to the non-Condon case but the transition dipole moment and, as explained above, the interaction Hamiltonian are no longer **R**-dependent. The overall dynamics is very similar to that observed in the non-Condon case, with the main difference being that the nodal line observed after the laser pulse on the ground-state nuclear wavepacket appears to be parallel to the *Y* axis rather than being tilted as in the non-Condon case. This feature can be clearly observed at times *t* = 10.2 and 31.5 fs in the S_0_ portion of the nuclear density (bottom panels of Figure 3).

It is worth commenting further on the development of the nodal line in the ground-state portion of the nuclear wave function after the laser pulse. This nodal line suggests the formation of a higher vibrational eigenstate in the electronic ground state in the Franck–Condon region. The asymmetry of the nuclear density in S_0_ observed at *t* = 31.5 fs results from the anharmonic nature of the S_0_ potential energy surface for the low vibrational states. From a more general perspective, this observation highlights something to keep in mind when analyzing a photochemical experiment because the outcomes of a light-induced process—photoproducts or hot ground-state molecules—are usually thought to originate from the dynamics in the excited electronic states following photoexcitation. Our observation underlines the possibility of forming vibrationally excited molecules without nonadiabatic processes simply as a result of the coupling with a laser pulse. However, nonadiabatic dynamics simulations are often initiated directly from the formed nuclear wavepacket in a given excited electronic state, neglecting the remaining ground-state nuclear amplitude. Although that is often an adequate approximation, the simple example presented here could indicate that the influence of a laser pulse on the contribution remaining in the ground electronic state may not always be negligible.

#### 3.1.2 Analysis of the Nuclear Dynamics

A more quantitative analysis of the photochemical dynamics described in section 3.1.1 can be obtained by monitoring the electronic population evolution and the formation of photoproducts (Figure 4).

In the top panel of Figure 4, we present the time evolution of the ground-state population for both the Condon and the non-Condon quantum dynamics simulations with, for reference, the strength of the external electric field over time. Initially, the population is fully in the ground state. Because of the coupling between the molecule and the laser pulse, the population starts to be transferred to the excited state just before 5 fs. Maximum values of around 64% (non-Condon) and 67% (Condon) of the population are excited to S_1_ at *t* = 10.2 fs, shortly after the laser pulse reaches its maximum intensity. The ground-state population subsequently plateaus at 48% (non-Condon) and 46% (Condon). After 26 fs, the excited-state population starts to decay back to the ground state as the S_1_ nuclear wavepacket reaches the conical intersection (section 3.1.1). This ground-state population reaches a final plateau at about 98% after about 39 fs. Overall, the evolution of the ground-state population is very similar in the non-Condon and Condon quantum dynamics simulations, with only minor quantitative differences emerging.

It is important at this stage to stress that the electronic population dynamics reported in the top panel of Figure 4 and discussed above is a representation-dependent quantity. In other words, this quantity is not (strictly speaking) an observable, and the assignment of an adiabatic electronic state is intrinsically linked to the Born–Oppenheimer picture. Hence, we complement our earlier observations with an analysis of the evolution of the nuclear density in the configuration space. To this end, we calculate the transmission probability through an ideal barrier parallel to the *Y* axis and containing the conical intersection along the reaction coordinate (along *X*). We propose that *X*_CI_, i.e., the position of the conical intersection in the *X* coordinate, delimits two regions: the photoreactant region (*X* < *X*_CI_) and the photoproduct region (*X* > *X*_CI_). Therefore, we can estimate the formation of photoproducts by integrating the nuclear density over all values of *Y* and for *X* > 3.86*a*_0_ (that is, *X* > *X*_CI_). In the case of a real photochemical experiment, the formation of photoproducts could be probed,^6^ and the passage through a conical intersection can open up pathways in the ground electronic state that would have been inaccessible by thermal evolution.

The transmission probability for the non-Condon and Condon cases is shown in the bottom panel of Figure 4. Within the Condon approximation, 54.2% of the population is transferred to the photoproduct region. In the non-Condon case, this value is slightly lower, reaching 52.0%. The transmission probability can be decomposed into ground- and excited-state contributions, going back to a Born–Oppenheimer picture. We observe that a majority of the photoproducts are formed in their ground states, 50.2% (non-Condon) and 52.3% (Condon). Hence, the slight differences between non-Condon and Condon excitations observed in the representation-dependent population dynamics are reflected in this “representation-free” observable.

### 3.2 Photochemical Experiment in the Exact-Factorization Picture

Now that we have discussed our in silico photochemical experiment from a Born–Oppenheimer perspective, we propose in the following section to reinterpret our findings using the tools offered by the exact-factorization picture. We recall that this representation proposes to replace the concepts of static potential energy surfaces associated with adiabatic electronic states and their nonadiabatic couplings with a single time-dependent potential energy surface (TDPES) and a time-dependent vector potential (TDVP). With the exact factorization, we move away from the representation of multiple electronic states visited by time-dependent nuclear wave functions and focus solely on a single nuclear wave function evolving under the influence of the single TDPES and TDVP.

**Figure 5:**
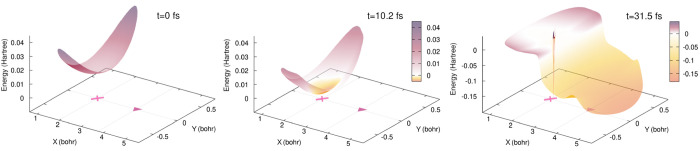
Schematic representation of the full time-dependent potential energy surface at three different times during the dynamics: 0, 10.2, and 31.5 fs. The positions of the Franck–Condon (magenta cross) point and conical intersection (burgundy triangle) are marked for reference. The color bar is given in hartrees (*E*_h_) and we note that its negative range has been extended for the *t* = 31.5 fs snapshot. The TDPES is represented only in the regions of nuclear configuration space where the total nuclear density is 10^–8^ or larger.

For the non-Condon dynamics, Figure 5 schematically depicts the behavior of the TDPES for the three same times along the dynamics as in the previous section, i.e., *t* = 0, 10.2, 31.5 fs. At time *t* = 0, the TDPES exhibits a single well in the Franck–Condon region, basically reproducing the shape of the S_0_ potential energy surface in this region (Figure 1). When the laser pulse is interacting with the molecule (*t* = 10.2 fs in Figure 5), the minimum in the TDPES decreases in energy and becomes asymmetrical around the Franck–Condon point. This distortion is due to the fact that the TDPES includes the effect of the time-dependent external field via *v*(**R**, *t*) (section 2.2). At later times (*t* = 31.5 fs in Figure 5), the TDPES develops a step separating the two portions of the nuclear wavepacket: one portion is localized in the Franck–Condon region, and the second one can be found in the vicinity of the conical intersection. In addition, the nodal line observed for the nuclear wave function in the Franck–Condon region is reflected in the TDPES as a potential barrier, reaching high positive and negative values. In the following section, we will provide a deeper analysis of the TDPES and its companion, the TDVP, for the studied photochemical experiment.

#### 3.2.1 Nuclear and Electronic Dynamics

As in section 3.1, we propose here to compare the quantum dynamics obtained with and without invoking the Condon approximation, but this time from an exact-factorization perspective.

**Figure 6:**
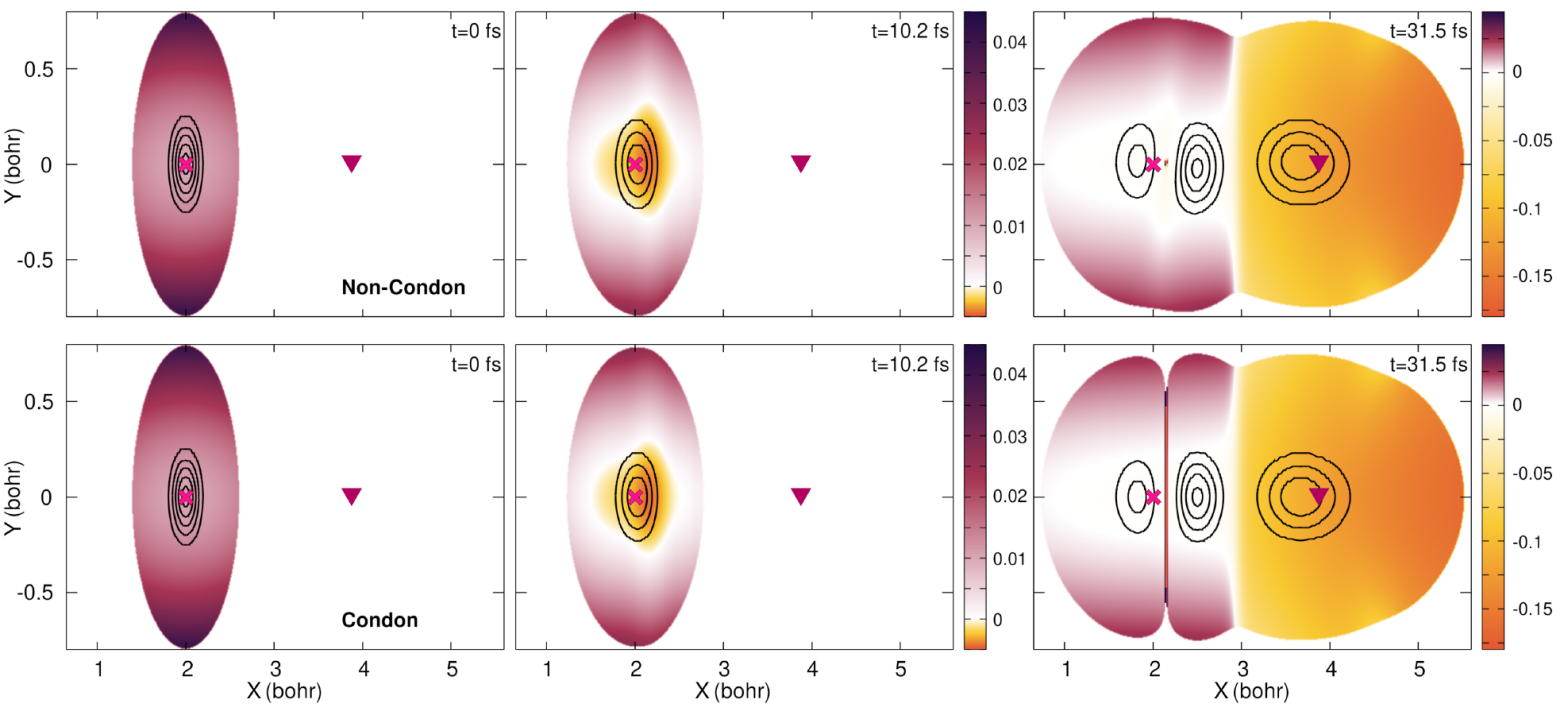
Time-dependent potential energy surface at three different times (0, 10.2, and 31.5 fs (from left to right)) for the non-Condon (top row) and Condon quantum dynamics (bottom row). The color bar is given in hartrees (*E*_h_). The nuclear density is superimposed (black contour lines). The positions of the Franck–Condon (magenta cross) point and conical intersection (burgundy triangle) are marked for reference.

Let us first focus on the evolution of the TDPES. In Figure 6, the full TDPES is plotted as a color map with superimposed black contour lines indicating the areas where the (full) nuclear density is mainly localized. At *t* = 0, both the non-Condon and the Condon simulations show an identical picture, where the TDPES shows a minimum around the center of the initial nuclear wave function and is curved upward to higher energies toward the borders, mainly reproducing the shape of the S_0_ potential energy surface (as discussed for Figure 5). When the laser pulse reaches its maximum intensity (at around *t* = 10.2 fs), the TDPES is lower in energy in the area just around the center of the nuclear wave function in comparison to the previously shown time step, where the yellow/orange areas correspond to negative energy values. At this time step, there appears to be no significant differences between the TDPES computed with and without the Condon approximation. Later in time, when the laser pulse is over (Figure 6, *t* = 31.5 fs), a step appears within the TDPES: the portion of the TDPES with *X* > 3*a*_0_ is significantly lower than that at *X* < 3*a*_0_. Furthermore, a marked difference between the non-Condon and Condon calculations emerges when looking at the TDPES in the region where the nodal line appears on the nuclear wave function around the FC position. In the non-Condon case, a localized peak can be observed between the two portions of the nuclear wave function, whereas a barrier forms within the Condon approximation, almost parallel to the *Y* axis and stretching all through the TDPES. It is worth stressing that the interesting features of the TDPES at *t* = 31.5 fs are all localized around the FC point and are caused by the formation of an eigenstate in this portion of the configuration. The TDPES in the region where a conical intersection would be observed in the Born–Oppenheimer picture is blatantly featureless, basically leading the nuclear wavepacket toward the photoproduct region (*X* > *X*_CI_).

**Figure 7:**
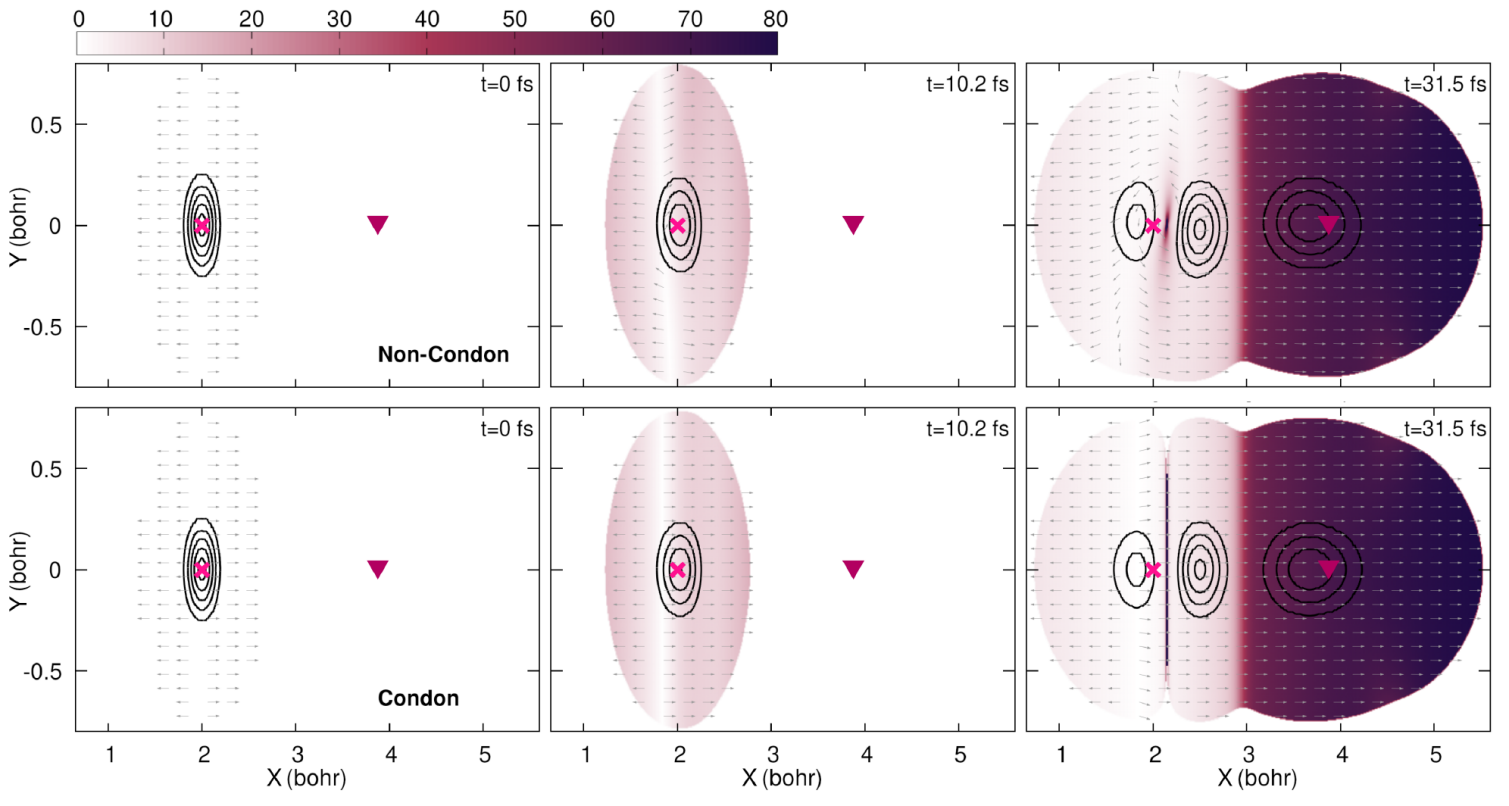
Time-dependent vector potential at three different times (0, 10.2, and 31.5 fs (from left to right)) for the non-Condon (top row) and Condon quantum dynamics (bottom row). The color map indicates the absolute value of the time-dependent vector potential in *ℏ*/*a*_0_, and the gray unit vectors show the orientation of the vector potential. The nuclear density is superimposed (black contour lines). The positions of the Franck–Condon (magenta cross) point and conical intersection (burgundy triangle) are marked for reference.

The TDPES is one of the time-dependent quantities of interest within the exact factorization. Another key ingredient of this formalism is the TDVP. We recall that the TDVP is equivalent to the nuclear momentum field within the chosen gauge, as detailed in section 2.2. Figure 7 shows the TDVP for the three critical times of the dynamics, with and without the Condon approximation. At time *t* = 0, the magnitude of the TDVP is very small. This can be understood from the definition of the initial condition for our dynamics, which is the ground vibrational state for the ground electronic state, at the FC point. From a quantum trajectory perspective, the momentum field corresponding to a nuclear (real) eigenstate would be zero everywhere.^44^ During the excitation, the TDVP increases in magnitude and triggers the dynamics of the nuclear wavepacket. Conversely with respect to our earlier observations with the TDPES, the TDVP has already developed some differences at *t* = 10.12 fs depending on the use of the Condon approximation. In the non-Condon simulation, the TDVP shows a non-negligible contribution along the *Y* direction, whereas within the Condon approximation, such a contribution is basically zero and the vector potential has components only along the *X* axis (see unit arrows in Figure 7). In both cases, however, one can observe an abrupt change in direction of the TDVP along the *X*-axis at around *X* = 1.9*a*_0_ for all values of *Y*. At *t* = 31.5 fs, the TDVP develops two clear portions. In the region of *X* > 3*a*_0_, the TDVP has an overall larger magnitude. In this region, the nuclear wavepacket is indeed pushed toward larger *X* values following the slope of the TDPES. Interestingly and consistent with our observation on the TDPES, this portion of the TDVP is smooth and does not reveal any features that would testify to a specific electron–nuclear coupling. This observation is particularly important if one considers that, in the Born–Oppenheimer picture, the nuclear wavepacket is passing through a conical intersection at this specific time (Figure 3). Such a behavior of the TDPES and TDVP simply highlights that the coupled electron–nuclear dynamics presented here should be seen as a non-event, with a molecule simply relaxing in energy, driven by the TDPES and TDVP. However, a Born–Oppenheimer picture enforces the description of this process with adiabatic electronic states, which are not representative of single electronic character. As a result, the S_1_ nuclear wavepacket in the Born–Oppenheimer picture suffers nearly singular nonadiabatic coupling to transfer to the S_0_ electronic state, hence preserving its electronic character. Conical intersections and singular nonadiabatic coupling terms therefore emanate from the intrinsic limitations posed by the adiabatic representation of the Born–Oppenheimer picture, and the exact factorization naturally remedies this issue by eliminating the notion of electronic states altogether.

Around the (FC) position, the TDVP has significantly smaller values at *t* = 31.5 fs, highlighting the rather stationary nature of the portion of the nuclear wave function localized in this region (Figure 7). In the region around *X* = 2.1*a*_0_ where the nuclear density splits, an intense localized peak appears on the TDVP in the non-Condon case while the Condon simulation leads to the appearance of an intense “delocalized” barrier parallel to the *Y* axis. In the non-Condon case, the unit vectors of the TDVP betray the fact that the nuclear wavepacket is somehow rotating around *X* = 2.1, *Y* = 0*a*_0_.

**Figure 8:**
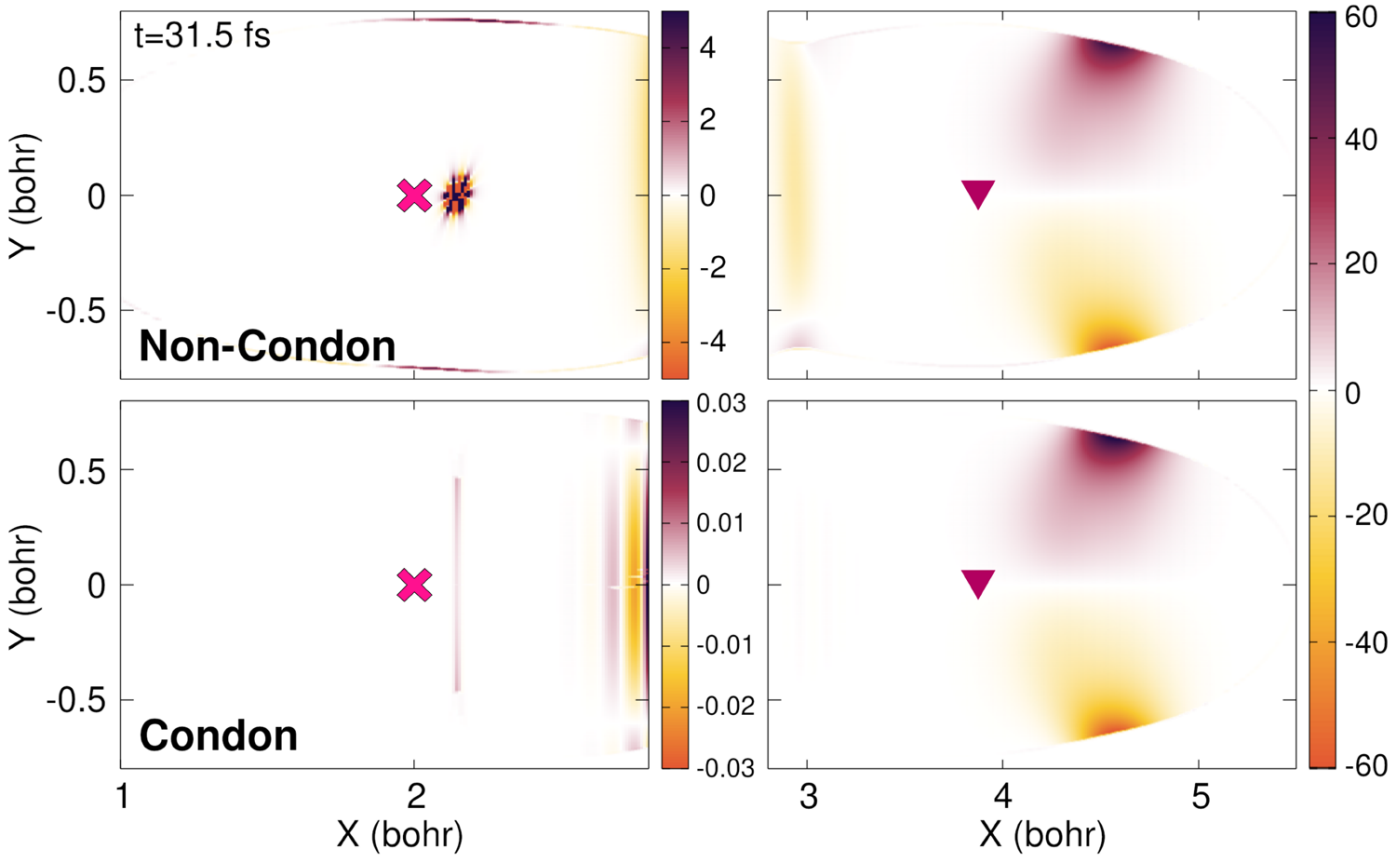
Curl of the TDVP at *t* = 31.5 fs for the non-Condon (top row) and Condon quantum dynamics (bottom row). The curl of the TDVP is given for the FC region *X* < 2.8*a*_0_ (left panels) as well as the CI and photoproduct regions *X* > 2.8*a*_0_ (right panels). The positions of the Franck–Condon (magenta cross) point and conical intersection (burgundy triangle) are marked for reference.

The TDVP is in general not irrotational,^30^ and the calculation of its line integral along a closed loop in nuclear (**R**) space yields a nonzero quantity. Such a quantity is a geometric phase whose value depends on the integration path. This dependence on the integration path means that this geometric phase is not quantized but is instead the case for the topological Berry phase arising in the post-Born–Oppenheimer picture in the presence of a conical intersection. In Figure 8, we prove numerically that the curl of the TDVP, i.e., [curl **A**(*X*, *Y*)]_*Z*_ = ∂_*X*_*A*_*Y*_(*X*, *Y*) – ∂_*Y*_*A*_*X*_(*X*, *Y*), is nonzero at time *t* = 31.5 fs in the non-Condon (upper panels) and Condon (lower panels) cases. Figure 8 gives the magnitude of the curl of the TDVP, which has only a *Z* component orthogonal to the *X*, *Y* plane in two dimensions. Because of the large difference in the curl magnitude, the plot for the FC region (*X* < 2.8*a*_0_, left panels) is separated from that for the CI region (*X* > 2.8*a*_0_, right panels). In line with our previous observations, the curl of the TDVP highlights the main differences between the non-Condon and Condon dynamics in the FC region. In the region where the ground-state wavepacket is located at the end of the laser pulse, the curl of the TDVP is nonzero only around *X* = 2.1, *Y* = 0*a*_0_ in the non-Condon case. The “rotating” behavior of the TDVP is encoded in the change of sign of its curl. (We checked numerically that this observed structure is robust (and not a numerical artifact) by varying the cutoff parameter for the calculation of the TDVP from 10^–6^ to 10^–10^.) The observed structure in the non-Condon case is lost in the Condon simulation, where the curl of the TDVP is nonzero and always positive only along a line at *X* = 2.1*a*_0_ parallel to the *Y* axis. The two simulations yield similar results in the CI region. In particular, the curl of the TDVP is zero at the CI. The change in curl sign between positive and negative values of *Y* attests from the overall spreading of the nuclear amplitude in this region.

**Figure 9:**
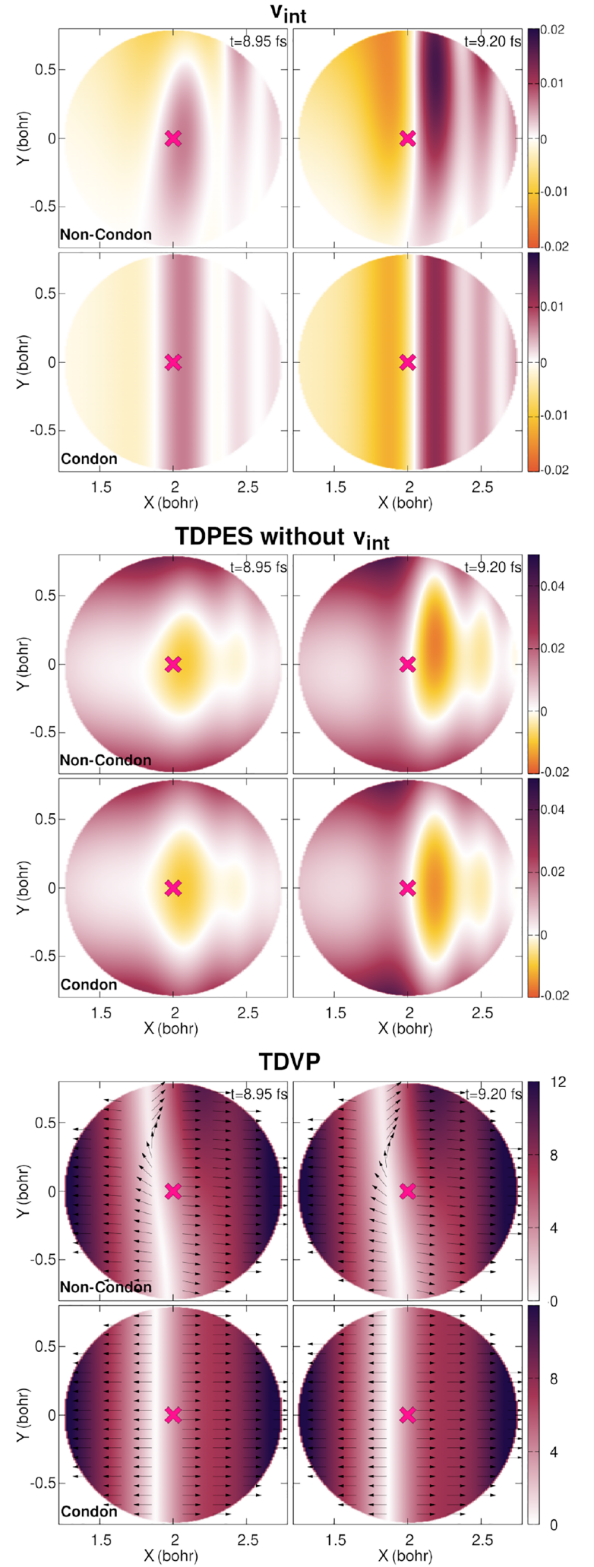
Time-dependent potential energy surface and vector potential during the laser pulse, for the non-Condon (top row of each panel) and Condon (bottom row of each panel) dynamics. Top panel: *v*_int_, contribution to the time-dependent potential energy surface coming from an external potential (here the interaction between the laser pulse and the molecule). Middle panel: time-dependent potential energy surface without the external potential. Bottom panel: time-dependent vector potential, where the color map indicates the absolute value and the black unit vectors show the orientation. The Franck–Condon point is indicated by a magenta cross in all plots. Color bars for the top and middle panels are given in hartrees (*E*_h_), and those of the lower panel are given in *ℏ*/*a*_0_.

To further unravel the differences observed in the TDPES and TDVP for the non-Condon versus Condon treatment of quantum dynamics, we decompose the TDPES into ϵ(**R**, *t*) and *v*_int_(**R**, *t*) at 2 times close to the maximum in the field envelop. The 8.95 fs time corresponds to a snapshot before the electric field of the laser pulse reaches a maximum while the other snapshot, *t* = 9.20 fs, is just after the maximum is passed. Figure 9 presents a close-up view of the TDPES and TDVP around the FC region for these two specific times. Looking only at the contribution to the TDPES coming from the coupling of the laser pulse to the molecule (*v*_int_(**R**, *t*) in eq 7 (*v*_int_, top panels in Figure 9)), we observe in the non-Condon case that an anisotropy along the *Y* direction is created, which is absent in the Condon approximation. This anisotropy is also visible in the term ϵ(**R**, *t*) (TDPES without *v*_int_, middle panels in Figure 9), where for instance the negative contributions of the TDPES are not symmetric along the *Y* direction in the non-Condon case. The variation of the TDPES caused by the interferences between the two components of the nuclear wave function is reminiscent of the observations of interference in nonadiabatic processes.^39^ Zooming in on the TDVP (bottom panels of Figure 9) highlights some additional interesting features. The TDVP already exhibits strong contributions along the *Y*-direction in the early stage of the dynamics in the non-Condon case, when the laser pulse is present, while in the Condon case no *Y*-contribution can be observed. The line along which the *X*-component of the TDVP changes sign is not parallel to the *Y*-axis in the non-Condon simulation, which is opposite to the Condon simulation.

The observations presented in this section show that the exact-factorization formalism offers an alternative perspective of a photochemical process to the conventional Born–Oppenheimer picture. The contribution to the molecular Hamiltonian coming from the light–matter interaction is fully encoded in the TDPES, and the evolution of the nuclear wavepacket is driven by the (single) TDPES and TDVP, which in turn encode the effect of the electronic dynamics on the nuclei. In the particular gauge chosen in this work, the TVDP is identified as the nuclear momentum field. Furthermore, we highlighted the combined effects of the TDPES and TDVP in inducing the splitting of the nuclear wavepacket into two portions in the Franck–Condon region, resembling the formation of a higher vibrational state of the electronic ground-state potential. Instead, the TDPES and TDVP are smooth where, in the Born–Oppenheimer picture, the nuclear wavepacket passes through a conical intersection and suffers singular nonadiabatic couplings.

The exact factorization and its time-dependent potential also highlight another interesting fact about photochemistry and photophysics. The presence of potential energy surfaces in a Born–Oppenheimer picture invites us to think of photochemical processes and chemical reactions in the ground electronic state in similar ways: by looking at these static potentials and possibly looking at their critical points and how they connect via minimum-energy paths. However, it is crucial to realize that such concepts are often not sufficient or valid in understanding a given photochemical process.^21^ In other words, a molecule does not have a defined photochemical reactivity per se, but such a photochemical reactivity depends on the initialization of the process (type of photoexcitation) and the following out-of-equilibrium dynamics on the coupled potential energy surfaces. The exact factorization highlights this fact by exhibiting different time-dependent potentials even for subtly different photochemical dynamics (here, simply employing or not employing the Condon approximation).

#### 3.2.2 Further Analysis of a Photochemical Process with Quantum and Classical Trajectories

Since the exact factorization encodes all of the coupled electron–nuclear dynamics in the TDPES and TDVP, this framework naturally lends itself to the propagation of nuclear trajectories, which can be used to further analyze and approximate the nuclear quantum dynamics without the need to introduce additional approximations. The challenge of propagating trajectories in a Born–Oppenheimer picture to describe nonadiabatic dynamics is notorious and caused by the presence of the lack of well-defined nuclear forces due to the presence of different (sometimes coupled) potential energy surfaces.^10,38^

As discussed in Appendix B, quantum trajectories can be propagated easily within the exact factorization by using the TDVP as a nuclear momentum field, integrating eq 25. Although the initial nuclear positions can be randomly sampled from the initial nuclear density, the initial nuclear momenta have to be chosen as **A**(**R**_0_, *t* = 0) (where **R**_0_ stands for the whole set of initial positions) because positions and momenta are not independent variables. We propagated 1000 quantum trajectories using the TDVP calculated at all times from the quantum dynamics simulations, without and with the Condon approximation.

**Figure 10:**
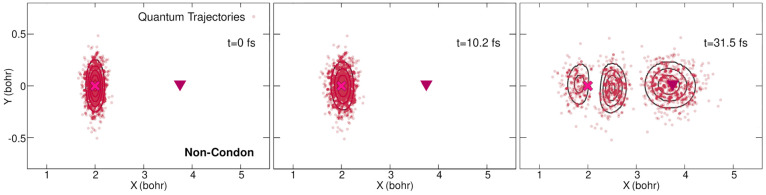
Positions of the quantum trajectories at three different times (0, 10.2, and 31.5 fs (from left to right)) for non-Condon dynamics. The full nuclear density from the quantum dynamics is superimposed (black contour lines). The positions of the Franck–Condon (magenta cross) point and conical intersection (burgundy triangle) are marked for reference.

As expected from their definition, the quantum trajectories closely follow the nuclear density of the quantum wavepacket at all three times as previously discussed (Figure 10). We show the results only for the non-Condon dynamics because the Condon dynamics are very similar. Several observations can be made by looking at the time evolution of the quantum trajectories in the non-Condon case (movies in the Supporting Information). First, we can see that the motion of the quantum trajectories is dominated by an evolution in the *X* direction. Second, the trajectories forming the two split portions of the nuclear wavepacket that remain in the vicinity of the FC region appear to rotate around a point close to the initial position of the nuclear wave function (but not exactly at the FC point due to the anharmonicity of the potential energy surface in this region). Such behaviors were predicted earlier on the basis of the analysis of the shape of the TDVP (section 3.2.1). Conversely, within the Condon approximation the quantum trajectories representing the nuclear wavepacket around the FC region do not significantly move once the splitting is complete. This behavior further supports the stationary nature of the driving wave function in this region and attests to the fact that the *Y* contribution of the momentum field is basically zero at later times in this region (as observed in Figure 7).

**Figure 11:**
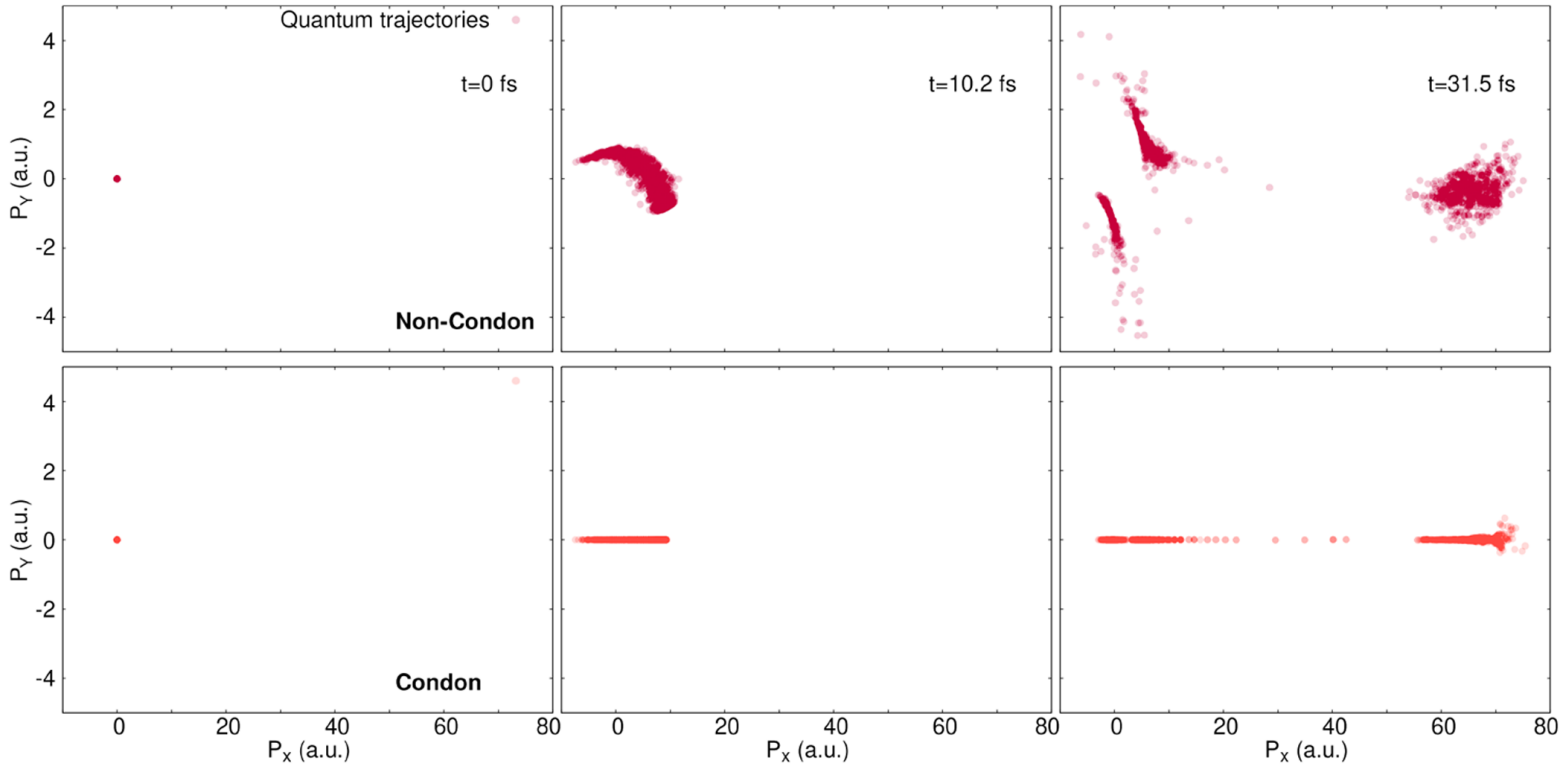
Components of the nuclear momentum in *X* (*P*_*X*_) and *Y* (*P*_*Y*_) for each quantum trajectory at three different times (0, 10.2, and 31.5 fs (from left to right)) for non-Condon (top row) and Condon (bottom row) dynamics. The label a.u. stands for *ℏ*/*a*_0_.

The distribution of the nuclear momenta for each quantum trajectory of the swarm highlights striking differences between the non-Condon and Condon dynamics at all times (Figure 11). At *t* = 0, the TDVP is very small in magnitude, i.e., close to zero, which is why the distribution of initial momenta appears to be highly localized around (0.0, 0.0). Subsequently, the quantum trajectories acquire larger momenta and start spreading to finally be distributed in three distinct areas by *t* = 31.5 fs in the non-Condon case (top right panel in Figure 11). The first group of trajectories shows large (positive) values for *P*_*X*_, between 60 and 80 *ℏa*_0_^–1^ and represents the photoproduct trajectories. Two groups of trajectories with comparably smaller *P*_*X*_ values spread along two lines, reaching a value of −4*ℏa*_0_^–1^ or 4*ℏa*_0_^–1^ for *P*_*Y*_. These last two groups depict the slow quantum trajectories in the region where a stationary state is formed. The distribution of nuclear momenta for the dynamics conducted within the Condon approximation looks significantly different from those of the non-Condon case described above (bottom row in Figure 11). At *t* = 10.2 fs, all of the trajectories have a dominant *X* contribution to their nuclear momenta. At *t* = 31.5 fs, a partitioning of the trajectories in momentum space is visible, as observed in the non-Condon case, forming two main groups: trajectories with a large *P*_*X*_ value and those with smaller momenta in the *X* direction. We note that the group with a smaller *P*_*X*_ contribution appears to be further split at the same position in *X* as in the non-Condon case. We also notice that a small portion of the trajectories with a very large *P*_*X*_ contribution at *t* = 31.5 fs also starts to spread along *P*_*Y*_.

**Figure 12:**
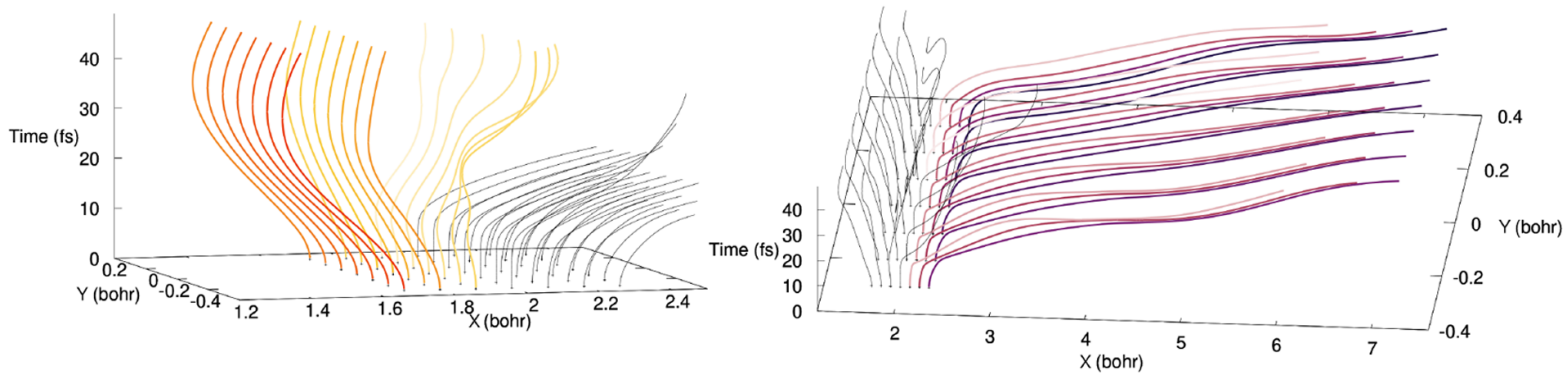
Traces of quantum trajectories in space and time for the non-Condon dynamics. These quantum trajectories were specially initialized on an equally spaced grid in configuration space to enhance clarity. The left panel shows in yellow/orange the trajectories remaining in the FC region. The right panel depicts in pink/purple the trajectories moving away from the FC region to reach the photoproduct region. The same trajectories are shown in both panels.

The fate of the quantum trajectories over time is made clearer by plotting their traces in time and space (Figure 12). We note that the quantum trajectories presented here were initialized from an equally spaced grid to improve the clarity of the plots in Figure 12. The trace representation of the quantum trajectories highlights the presence of two groups of quantum trajectories: those that remain that evolve around the FC region (left panel of Figure 12) and those that leave that translate the formation of photoproducts (right panel of Figure 12). Interestingly, it appears from this analysis that quantum trajectories with a larger *X* value in the FC region are more likely to follow the photoproduct path than those started at *t* = 0 fs at a smaller value of *X*. This trace representation for the quantum trajectories also offers an opportunity to show that they do not cross in configuration space.^45^ This noncrossing rule is a strict requirement for quantum trajectories and a direct consequence of the single-valuedness of the nuclear wave function.

An interesting feature of quantum trajectories is that they can easily be transformed into their more classical analogues by neglecting the so-called quantum potential, which acts as a nonlocal glue for the trajectories (Appendix B). We can therefore propagate classical trajectories by using the TDPES and TDVP computed from the quantum wavepacket dynamics, but with the removal of the contribution from the quantum potential *Q*_pot_(**R**, *t*). Hence, the classical trajectories, initialized from 1000 nuclear positions and momenta sampled from a Wigner distribution, come directly from the integration of eqs 27 and 28.

**Figure 13:**
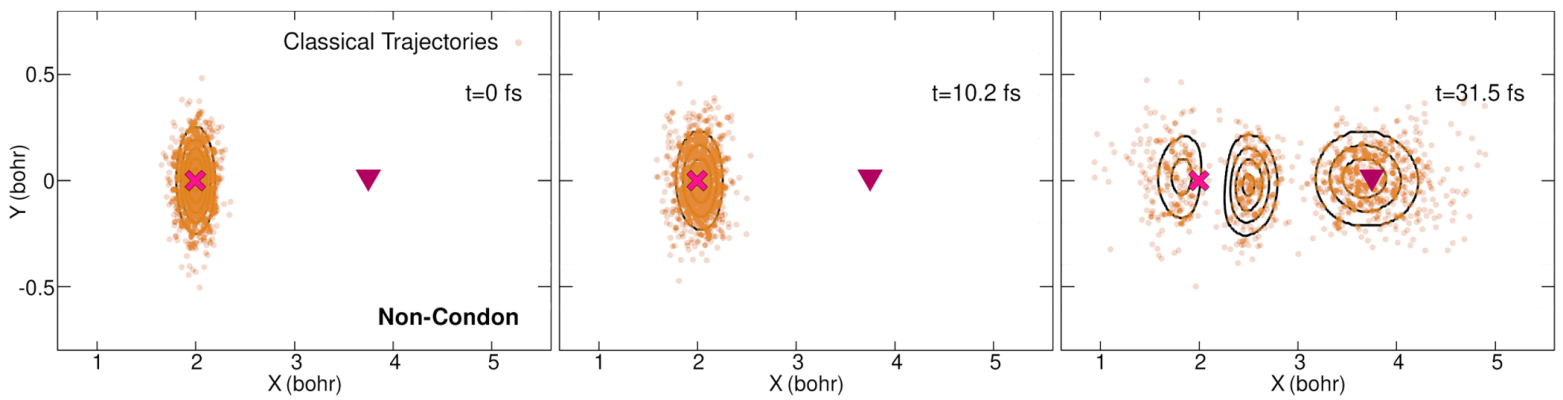
Positions of the classical trajectories at three different times (0, 10.2, and 31.5 fs (from left to right)) for non-Condon dynamics. The full nuclear density from the quantum dynamics is superimposed (black contour lines). The positions of the Franck–Condon (magenta cross) point and conical intersection (burgundy triangle) are marked for reference.

The positions of these classical trajectories at *t* = 0, 10.2, 31.5 fs show rather good agreement with the nuclear wavepacket (Figure 13), even if it is clear by comparison with Figure 10 that the classical trajectories spread more in both the *X* and *Y* directions over time. (We note that the same initial positions were used for the classical and quantum trajectories.) Looking at the full evolution of the classical trajectories over time further reinforces this observation (movies in the Supporting Information), with trajectories exhibiting important oscillations along the *Y* coordinate.

**Figure 14:**
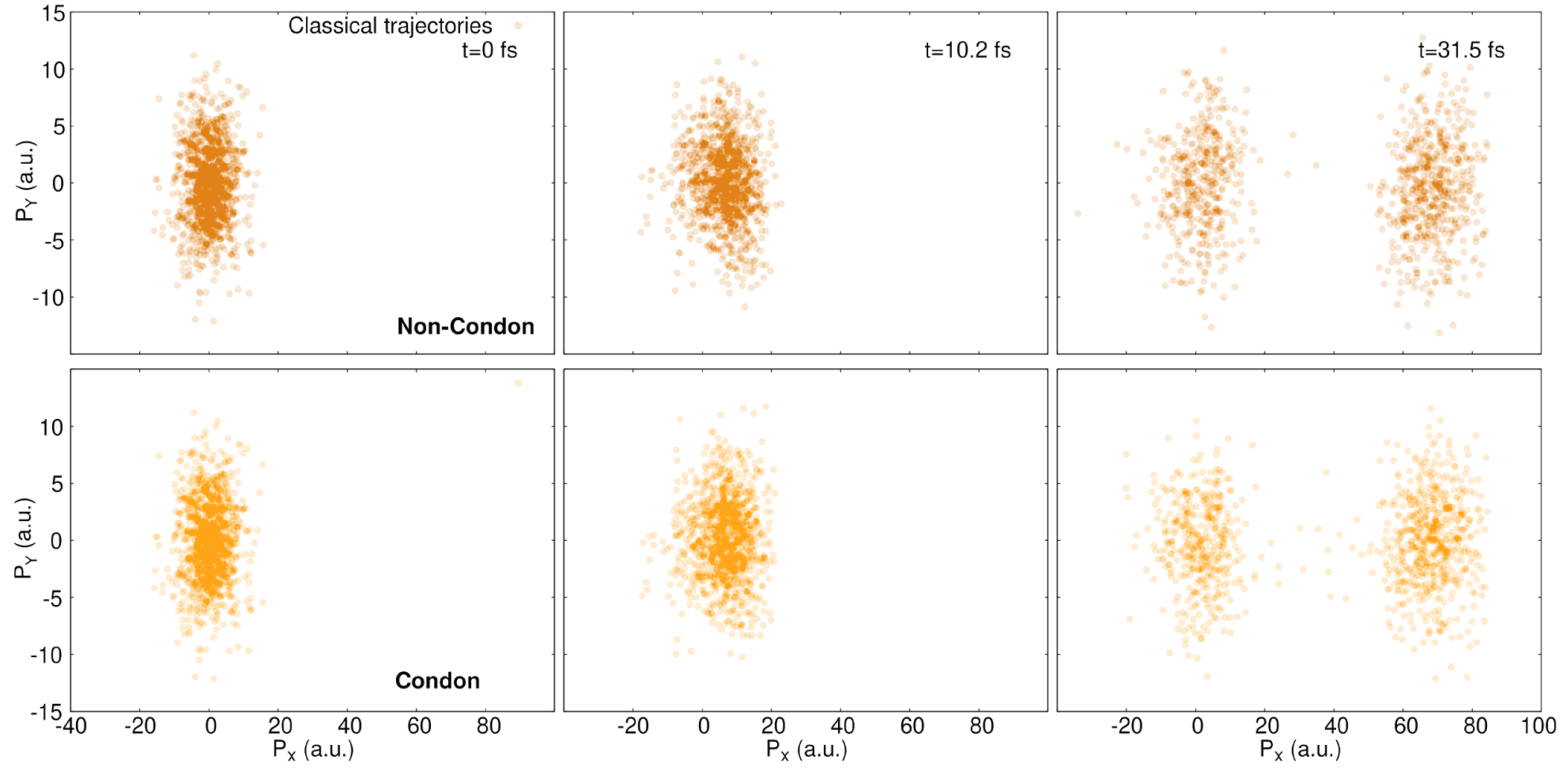
Components of the nuclear momentum in *X* (*P*_*X*_) and *Y* (*P*_*Y*_) for each classical trajectory at three different times (0, 10.2, and 31.5 fs (from left to right)) for non-Condon (top row) and Condon (bottom row) dynamics. The label a.u. stands for *ℏ*/*a*_0_.

The projection of the classical trajectories in nuclear momentum space for the three selected times (Figure 14) is strikingly different from that of the quantum trajectories discussed earlier (Figure 11). The distribution of nuclear momenta for the classical trajectories is spread at all times and does not exhibit the peculiar structure observed for the quantum trajectories. The magnitude of the nuclear momenta is also significantly larger in the *X* direction and, more specifically, in the *Y* direction. This observation explains the oscillating behavior in the *Y* coordinate described above for the classical trajectories. At *t* = 31.5 fs, the momenta distribution separates into only two regions (Figure 14): one part with momenta distributed around *P*_*X*_ = 0 and the other part with momenta distributed around *P*_*X*_ = 70*ℏa*_0_^–1^. Interestingly, the distribution of classical nuclear momenta in the non-Condon case closely resembles that in the Condon case, as if the removal of the quantum potential washed out the fine differences observed during the formation of the stationary state.

**Figure 15:**
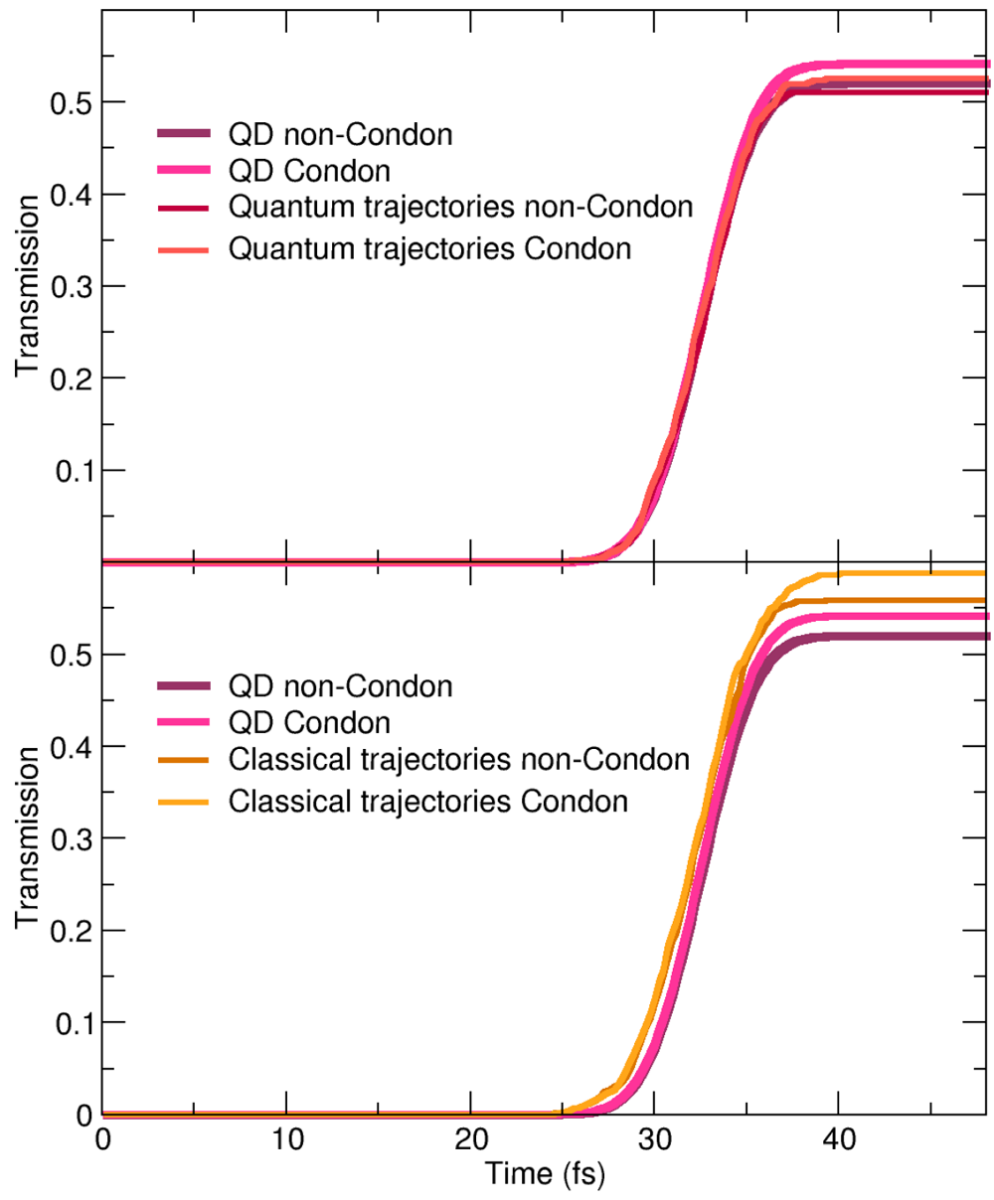
Time trace of the transmission probabilities through the line defined by *X* > 3.86*a*_0_ obtained for the quantum (top panel, red lines) and classical trajectories (bottom panel, orange lines) in the non-Condon and Condon cases. The results from quantum dynamics (QD) are given for reference (non-Condon with purple lines and Condon with magenta lines).

We conclude this analysis by calculating the transmission toward photoproducts, as performed in section 3.1.2 but here based on the distribution of the quantum and classical trajectories over time. To this end, we simply count the trajectories with *X* > 3.86*a*_0_ (as described in section 3.1.2) for both quantum and classical trajectories and in non-Condon and Condon dynamics. Quantum trajectories appear to slightly underestimate the reference transmission probability (from the quantum dynamics) by around 0.01 for the non-Condon dynamics and 0.02 for the Condon dynamics (top panel of Figure 15). The classical trajectories overestimate the transmission probabilities in both non-Condon and Condon cases by 0.04 and 0.05, respectively (bottom panel of Figure 15). This deviation is marginal, and both quantum and classical trajectory-based dynamics reproduce reasonably well the qualitative evolution of the nuclear density while providing quantitatively good estimates for the transmission probability, an observable that would, for a real molecule, connect to the formation of photoproducts and thus to the quantum yield of a photochemical reaction.

One important aspect that needs to be stressed at this point is the fact that both quantum and classical trajectories benefitted from the definition of the time-dependent potentials used to propagate them thanks to the formalism of the exact factorization (and within our choice of gauge). More specifically, simulating a full photochemical experiment as done here using quantum or classical trajectories within a Born–Oppenheimer picture would have made the simulations dramatically more complex. Transfers, hops, averaging, or spawns would have to be invoked to allow the trajectories to visit different electronic states due to the influence of a laser pulse or nonadiabatic transitions. The exact factorization simplifies that by providing the single TDPES and TDVP.

## 4 Conclusions

We introduced in this work a comparison between the Born–Oppenheimer and exact-factorization picture of an in silico photochemical experiment, from the initial photoexcitation with an ultrashort laser pulse to the formation of photoproducts. Our extensive analysis of the subsequent photodynamics revealed how to picture a photochemical reaction in the exact factorization formalism and how its quantities, namely, the time-dependent vector and scalar potentials, behave when an external laser pulse shakes the electronic wave function and triggers the formation of a nuclear wavepacket. As a side product of the excitation with a laser pulse, we could also observe the formation of a stationary state in the Franck–Condon region and the corresponding behavior of the time-dependent potentials. The Condon approximation can alter the dynamics of the nuclear wavepacket, even if the formation of photoproducts, which is a representation-free quantity, does not suffer from this approximation in the presented model. Interestingly, the passage through a conical intersection in the Born–Oppenheimer picture becomes a nonevent in the exact factorization: the singularities and degeneracies at conical intersections are in stark contrast with the featureless time-dependent potentials of the exact factorization. Finally, we highlighted one of the exciting features of the exact factorization in the context of a full photochemical process: the possibility to naturally introduce trajectories as an approximation for the nuclear dynamics. The propagation of these trajectories is trivial because the exact factorization has only a single time-dependent vector and scalar potential, meaning that no hops or spawns are required to describe regions of strong nonadiabaticity. Distinguishing between classical and quantum trajectories, we discussed the suitability of using trajectories to simulate photochemical processes with the exact factorization as well as the importance of properly selecting initial conditions.

## Appendix A: Expressing the TDVP and TDPES within a Diabatic Basis

Quantum mechanics allows one to switch from the Dirac representation |ψ⟩ of a state to the wave function representation ψ(**r**) with the operation ⟨**r**|ψ⟩ = ψ(**r**). The same holds true for the exact-factorization quantities: the electronic-state vector at time *t*, |Φ(*t*; **R**)⟩, which parametrically depends on **R**, is related to the conditional wave function of eq 6 as ⟨**r**|Φ(*t*; **R**)⟩ = Φ(**r**, *t*; **R**). Since the electronic Hamiltonian (eq 9) is given in the diabatic basis, we represent in our calculations the state |Φ(*t*; **R**)⟩ in such a basis and identify the expansion coefficients (or diabatic nuclear amplitudes) as χ_*l*_^(*d*)^(**R**, *t*). All quantities analyzed in this work, namely, the nuclear density, the TDPES, and the TDVP, are obtained by integrating over the electronic degrees of freedom, thus by summing over the diabatic states. We give their expressions below.

The nuclear density |χ(**R**, *t*)|^2^ is simply

13

The explicit expression of the TDPES is

14

and can thus be decomposed as the sum of four terms ϵ(**R**, *t*) = ϵ_GI1_(**R**, *t*) + ϵ_GI2_(**R**, *t*) + ϵ_GI3_(**R**, *t*) + ϵ_GD_(**R**, *t*), with the first three being gauge-invariant (GI) and the last one being gauge-dependent (GD) under the (gauge) transformations discussed in section 2.2. Transforming the integration over **r** to the sum over the diabatic states, considering that the nonadiabatic coupling vectors are identically zero in this basis, and using the notation for the matrix elements of the electronic Hamiltonian introduced in eq 9, the terms in the expression of the TDPES are

15

16

17

18

Similarly, the TDVP is given by

19

While the TDPES and TDVP can be expressed in the diabatic basis, as done above, it is critical to note that they *do not* depend on any particular choice of electronic representation.

## Appendix B: Trajectory-Based Solution of the Nuclear Exact Factorization Equation

Because the coupled nuclear and electronic equations of motion give rise to a single time-dependent vector potential and a single time-dependent potential energy surface in the exact factorization, one intriguing question is whether one could simplify the nuclear dynamics by representing the nuclear probability density with trajectories evolving on the basis of the two potentials. In previous work,^35,43^ it was shown that by inserting the polar form of the nuclear wave function, , into the time-dependent Schrödinger equation, one can derive an evolution equation for the phase that can furthermore be identified as a (nuclear) Hamilton–Jacobi equation (with ):

20

The last term on the right-hand side is the so-called quantum potential, *Q*_pot_(**R**, *t*), which is responsible for an important portion of the nuclear quantum effects. The term containing the nuclear momentum field, **P**_ν_(**R**, *t*) = **P̃**_ν_(**R**, *t*) + **A**_ν_(**R**, *t*), is the nuclear kinetic energy, and the sum ϵ(**R**, *t*) + *v*_int_(**R**, *t*) + *Q*_pot_(**R**, *t*) represents the potential energy. As discussed in an earlier work,^43^ this Hamilton–Jacobi partial differential equation can be solved by using the method of characteristics. This results in Hamilton-like evolution equations (which are ordinary differential equations to be solved for an a priori infinite number of initial conditions)

21

22

or equivalently

23

24

for the trajectories , with corresponding momenta .

Enforcing our choice of gauge for this work (*S*(**R**, *t*) = 0, see section 2.2) in the characteristic representation of the nuclear dynamics, we find that eqs 21 and 22 simplify to

25

26

proving that the time-dependent vector potential fully accounts for the nuclear momenta of the trajectories. It is worth noting that, within the exact factorization, the nuclear momentum field computed with Ψ(**r**, **R**, *t*) is the sum of a curl-free term—the gradient of *S*(**R**, *t*)—and a (in general not irrotational) contribution due to the time-dependent vector potential, as shown in ref (38). With our choice of gauge, the curl-free contribution from the gradient of *S*(**R**, *t*) becomes zero. In Appendix C, we prove analytically the identity given in eq 26 and show that, in the chosen gauge, .

Propagating trajectories solely using either eq 25 or coupled eqs 23 and 24 yields what we refer to as quantum trajectories. In particular, eq 25 corresponds to the Bohmian definition of the velocity field for quantum-mechanical particles described by a real wave function in the presence of an external vector potential.^46^ By neglecting the quantum potential in *H*_*n*_(**P**(*t*), **R**(*t*), *t*), the nuclear Hamiltonian becomes classical, i.e., *H*_*n*_^cl^(**P**(*t*), **R**(*t*), *t*). This classical approximation is used when integrating eqs 23 and 24 to generate classical trajectories. The corresponding evolution equations for the classical trajectories then read

27

28

## Appendix C: Nuclear Forces for the Quantum Trajectories

In this appendix, we prove eq 26 of Appendix B by demonstrating that, within the chosen gauge, *S*(**R**, *t*) = 0, .

The nuclear Hamiltonian identified in the Hamilton–Jacobi equation in eq 20 reads

29

when imposing the gauge as **P** ≈ 0. From the definition of the time-dependent scalar potential ϵ(**R**, *t*) given in section 2.2 and in eq 14, we identify ϵ_BO_(**R**, *t*) = ϵ_GI1_(**R**, *t*) and ϵ_bBO_(**R**, *t*) = ϵ_GI2_(**R**, *t*) such that the full nuclear Hamiltonian reads

30

It is easy to prove that while ϵ_BO_(**R**, *t*) (Born–Oppenheimer term) and ϵ_bBO_(**R**, *t*) (beyond-Born–Oppenheimer term) are gauge-invariant contributions because they remain unchanged if the electronic wave function is modified by a phase factor that depends only on **R** and *t*, ϵ_GD_(**R**, *t*) is gauge-dependent (GD).^38^ Furthermore, it might seem that in the chosen gauge the nuclear Hamiltonian is purely potential energy. This is not actually the case because it has been shown in refs (29) and (47) that ϵ_bBO_(**R**, *t*) contributes to the nuclear kinetic energy despite appearing as a scalar potential included in the expression of ϵ(**R**, *t*).

With the aim of manipulating previous equations, we use the definition of the electronic wave function as . This expression is inserted into the definitions of the ϵ_BO_(**R**, *t*) and ϵ_GD_(**R**, *t*) potentials. In addition, we write explicitly the expectation values as integrals over the electronic configuration space such that

31

32

The second term in ϵ_GD_(**R**, *t*), being purely imaginary, is clearly identically zero because the whole expression is purely real.

The contribution to the time-dependent scalar potential arising from the external field, i.e., *v*_int_(**R**, *t*), can be rewritten as well as

33

The “kinetic” part of the time-dependent scalar potential, ϵ_bBO_(**R**, *t*), becomes

34

Here, we used the chain rule for the derivative with respect to **R**_ν_, namely, . Applying once more the chain rule to the first term on the right-hand side of eq 34 yields

35

Using the partial normalization condition, ∫ d**r**|Φ(**r**, *t*; **R**)|^2^ = 1∀**R**, *t*, to show that the first term on the right-hand side of eq 35 is identically zero implies that

36

proving that eq 36 is purely imaginary. However, ϵ_bBO_(**R**, *t*) is real overall, which implies that such a purely imaginary contribution, from the first integral in its definition (eq 34), should be canceled out exactly by the imaginary part of its second term. As a result, we can rewrite eq 34 as

37

Summarizing the results obtained so far, we can rewrite the nuclear Hamiltonian as

38

Note that the first integral of eq 38 can be simplified using the time-dependent Schrödinger equation. In fact, the operator in parentheses equals the negative of the nuclear kinetic energy, i.e., , and the whole integral is the negative of the expectation value of *T̂*_n_(**R**) over the molecular wave function. Hence, we can now define the condition for (the nuclear gradient of) *H*_*n*_(**R**, *t*) to be equal to zero:

39

We use once again the definition of Φ(**r**, *t*; **R**) in terms of the molecular and nuclear wave functions, and we apply  on the left-hand side of eq 39 such that

40

Therefore, we see that the condition given by eq 39 would be fulfilled if, by taking the real part of eq 40, the last two terms would cancel out.

To show that, let us investigate the real part of the contribution depending on the full molecular wave function, where we use Ψ(**r**, **R**, *t*) = Φ(**r**, *t*; **R**)|χ(**R**, *t*)|, namely,

41

The first term on the right-hand side of eq 41 is identically zero because it is purely imaginary (eq 36); the integral in the second term yields 1 thanks to the partial normalization condition. As a result, we have

42

proving that when taking the real part of the last two terms of eq 40, they exactly cancel out. Therefore, the condition posed by eq 39 is fulfilled, as stated above.

Finally we can show that the nuclear Hamiltonian of eq 38 is zero in the chosen gauge

43

meaning that its gradient is zero as well.

## Supporting Information Available

Movie of the classical and quantum trajectories without invoking the Condon approximation (MP4)
Movie of the classical and quantum trajectories with invoking the Condon approximation (MP4)
Movie of the classical and quantum trajectories without invoking the Condon approximation (MP4)
Movie of the classical and quantum trajectories with invoking the Condon approximation (MP4)
Description of the movies (PDF)
