# Supplementary material for: A Photochemical Reaction in Different Theoretical Representations
Source: J Phys Chem A. 2022 Feb 14;126(7):1263–81. doi: 10.1021/acs.jpca.1c09604 (PMC8883471; doi:10.1021/acs.jpca.1c09604)
Supplement: Supplementary file 5 — jp1c09604_si_005.pdf [file jp1c09604_si_005.pdf]

A photochemical reaction in different theoretical representations

Lea M. Ibele,[†] Basile F. E. Curchod,[*,†] and Federica Agostini[*,‡]

†*Department of Chemistry, Durham University, Durham DH1 3LE, United Kingdom*

‡*Université Paris-Saclay, CNRS, Institut de Chimie Physique UMR8000, 91405 Orsay, France*

E-mail: basile.f.curchod@durham.ac.uk; federica.agostini@universite-paris-saclay.fr

As Supporting Information we include four animations describing the time evolution of the classical and quantum trajectories described in the main text with and without the Condon approximation. The red lines show the positions of the trajectories in the X, Y-plane at all times and the black dots are the instantaneous positions of the trajectories (each following the corresponding red line).
